# Gene-expression profiling of individuals resilient to Alzheimer's disease reveals higher expression of genes related to metallothionein and mitochondrial processes and no changes in the unfolded protein response

**DOI:** 10.1186/s40478-024-01760-9

**Published:** 2024-04-25

**Authors:** Luuk E. de Vries, Aldo Jongejan, Jennifer Monteiro Fortes, Rawien Balesar, Annemieke J. M. Rozemuller, Perry D. Moerland, Inge Huitinga, Dick F. Swaab, Joost Verhaagen

**Affiliations:** 1 Department of Neuroregeneration, Netherlands Institute for Neuroscience, Royal Netherlands Academy of Arts and Sciences, Meibergdreef 47, 1105 BA Amsterdam, The Netherlands; 2 Amsterdam UMC Location University of Amsterdam, Epidemiology and Data Science, Meibergdreef 9, 1105 AZ Amsterdam, The Netherlands; 3 Amsterdam Public Health, Methodology, Amsterdam, The Netherlands; 4 Amsterdam Infection and Immunity, Inflammatory Diseases, Amsterdam, The Netherlands; 5 Department of Neuropsychiatric Disorders, Netherlands Institute for Neuroscience, Institute of the Royal Netherlands Academy of Arts and Sciences, Meibergdreef 47, 1105 BA Amsterdam, The Netherlands; 6 Department of Pathology, Amsterdam Neuroscience, Amsterdam UMC - Location VUmc, Amsterdam, The Netherlands; 7 Department of Neuroimmunology, Netherlands Institute for Neuroscience, Institute of the Royal Netherlands Academy of Arts and Sciences, Meibergdreef 47, 1105 BA Amsterdam, The Netherlands; 8 Center for Neuroscience, Swammerdam Institute for Life Sciences, University of Amsterdam, Amsterdam, The Netherlands; 9 Center for Neurogenomics and Cognitive Research, Neuroscience Campus Amsterdam, VU University, Boelelaan 1085, 1081 HV Amsterdam, The Netherlands

**Keywords:** Alzheimer’s disease, Resilience, Post-mortem tissue, RNA-sequencing, Metallothionein, Mitochondria, Unfolded protein response

## Abstract

Some individuals show a discrepancy between cognition and the amount of neuropathological changes characteristic for Alzheimer’s disease (AD). This phenomenon has been referred to as ‘resilience’. The molecular and cellular underpinnings of resilience remain poorly understood. To obtain an unbiased understanding of the molecular changes underlying resilience, we investigated global changes in gene expression in the superior frontal gyrus of a cohort of cognitively and pathologically well-defined AD patients, resilient individuals and age-matched controls (n = 11–12 per group). 897 genes were significantly altered between AD and control, 1121 between resilient and control and 6 between resilient and AD. Gene set enrichment analysis (GSEA) revealed that the expression of metallothionein (MT) and of genes related to mitochondrial processes was higher in the resilient donors. Weighted gene co-expression network analysis (WGCNA) identified gene modules related to the unfolded protein response, mitochondrial processes and synaptic signaling to be differentially associated with resilience or dementia. As changes in MT, mitochondria, heat shock proteins and the unfolded protein response (UPR) were the most pronounced changes in the GSEA and/or WGCNA, immunohistochemistry was used to further validate these processes. MT was significantly increased in astrocytes in resilient individuals. A higher proportion of the mitochondrial gene MT-CO1 was detected outside the cell body versus inside the cell body in the resilient compared to the control group and there were higher levels of heat shock protein 70 (HSP70) and X-box-binding protein 1 spliced (XBP1s), two proteins related to heat shock proteins and the UPR, in the AD donors. Finally, we show evidence for putative sex-specific alterations in resilience, including gene expression differences related to autophagy in females compared to males. Taken together, these results show possible mechanisms involving MTs, mitochondrial processes and the UPR by which individuals might maintain cognition despite the presence of AD pathology.

**Supplementary Information:**

The online version contains supplementary material available at 10.1186/s40478-024-01760-9.

## Introduction

Alzheimer disease (AD) is the most common form of dementia, affecting 47 million individuals worldwide [1]. The presence of the classical neuropathological hallmarks of AD, amyloid beta (Aβ) plaques and aggregation of hyper-phosphorylated tau (pTau) in tangles, is required for a final diagnosis. However, it has been estimated that up to 30% of cognitively intact elderly harbor significant amounts of AD neuropathology [2]. Thus, a considerable number of individuals have a discrepancy between cognition and pathology, indicating that AD neuropathology itself might not be enough to explain cognitive decline. The reason why these individuals remain cognitively intact is currently poorly understood. This phenomenon has been labeled as ‘reserve’ or ‘resilience’ [3] and represents an interesting subject to study, as understanding the molecular and cellular mechanisms underlying resilience could lead to novel therapeutic avenues.

While the exact molecular and cellular underpinnings of resilience remain to be elucidated, several studies have demonstrated alterations in post-mortem brain tissue of resilient donors, including changes in synaptic proteins, glial cells and the amount of AD pathology. In particular, changes in the amount of pre- and post-synaptic proteins such as SNARE proteins, synaptophysin and elongated dendritic spines were found in resilient individuals [4–6]. Others showed either reduced numbers of activated microglia or hyperactive microglia near plaques, based on activation markers such as CD68 [5, 7, 8]. Importantly, while it was hypothesized that resilient donors have a similar progression of AD pathology but are able to stay cognitively intact for a longer period, lower amounts of oligomeric Aβ and pTau have been shown in resilient donors [5, 9, 10]. Furthermore, lower levels of other pathological inclusions such as Lewy bodies (LBs) or TAR DNA-binding protein 43 (TPD-43), often present in AD donors, were found in resilient donors [11, 12]. More recently, several studies have used RNA sequencing to study the molecular basis of resilience. This has led to specific targets such as the transcription factor MADS box transcription enhancer factor 2 (MEF2C) in excitatory neurons [13] and to the observation of an increased expression of genes involved in synaptic and mitochondrial function [14]. RNA sequencing has also resulted in the observation that clusters of cells can respond differently to AD pathology in cognitively intact donors with a low pTau load compared to AD patients with a high pTau load [15].

In order to further elucidate how resilient individuals can remain cognitively intact, we have identified a cohort of individuals with intact cognition and significant amounts of AD neuropathology in the brain collection of the Netherlands Brain Bank (NBB), herein after labeled as ‘resilient’. To identify potential mechanisms related to resilience, we investigated changes in gene expression in the superior frontal gyrus (SFG) using RNA-sequencing and compared the resilient donors to demented AD patients and cognitively intact age-matched controls. The prefrontal cortex was used as it is an important region for executive functions, including working memory and cognitive flexibility [16], which are both impaired in AD. To further establish how our findings relate to resilience, we also quantified and controlled for the amount of AD neuropathology in the same brain region to investigate if changes in gene expression are driven by the amount of local pathology or may provide resilience despite the amount of pathology. We have validated mechanisms of interest by immunohistochemistry (IHC). Finally, we examined possible sex-dependent resilience mechanisms.

## Material and methods

### Human brain tissue

Brain donors were selected from the NBB or from the 100+ study [17]. Informed consent for a brain autopsy and for the use of the brain material and clinical data for research purposes was obtained by the NBB according to international ethical guidelines. Autopsy procedures were approved by the Medical Ethic Committee of the VU Medical Center, Amsterdam, the Netherlands. The autopsy and neuropathological assessment were performed using standardized protocols, including neuropathological classification according to Braak [18], CERAD [19] and the National Institute on Aging–Alzheimer’s Association guidelines [20], cerebrovascular diseases, Lewy bodies (LBs), hippocampal sclerosis (HS), limbic-predominant age-related (LATE) TDP-43 and ubiquitin (P62). Furthermore, a clinical summary including a clinical dementia rating (CDR) or global deterioration scale (GDS) was available for each donor. Either the CDR or GDS was used to retrospectively determine the cognition up to 3 months prior to death, which was performed by a clinical specialist or general practitioner.

### DNA isolations and genotyping

DNA was isolated from 50 mg of tissue from the SFG or cerebellum using the DNeasy Blood & Tissue Kit (Qiagen, Valencia, CA, USA). Tissue was lysed over night at 56 °C after which the lysate was filtered over DNeasy Mini Spin Columns (Qiagen, Valencia, CA, USA) and further processed according to manufacturer’s instructions. Apolipoprotein E (ApoE) genotype was determined with the TIB MOLBIOL LightMix Kit APOE C112R R158C with a LightCycler® 480 System and hybridization probe method.

### Donor inclusion

**Fig. 1:**
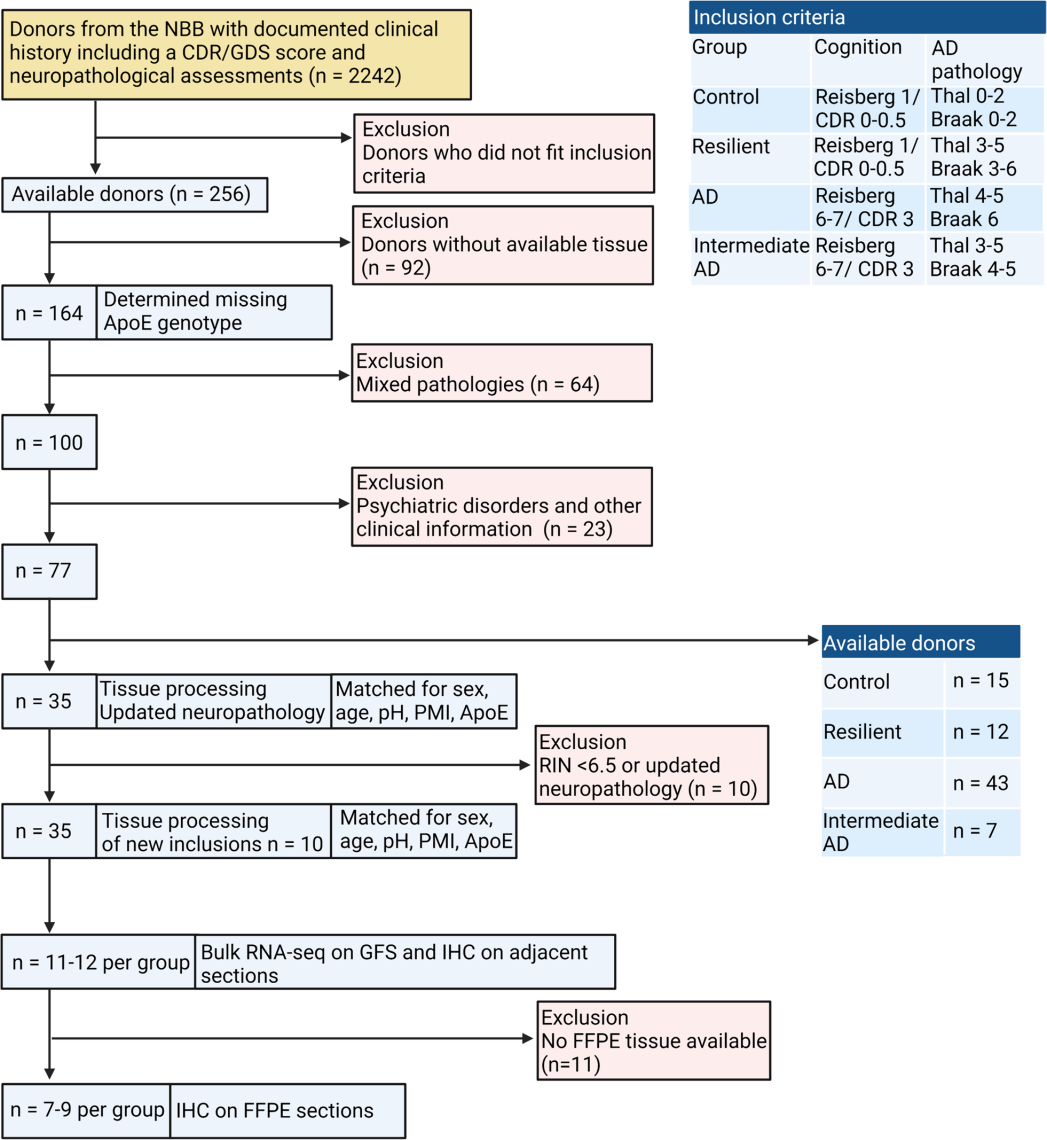
Flow diagram of the donor selection from the NBB and performed experiments. The selection of donors involved a step by step process aiming to include (depicted in light blue) or exclude (depicted in light red) donors. From the brain collection from the NBB, donors were selected fitting our inclusion criteria. Donors without available frozen tissue of the superior frontal gyrus (SFG), donors with mixed neuropathologies from other disease like multiple sclerosis, but also donors with high amounts of comorbid pathology such as TPD-43 or cortical LBs and donors with psychiatric disorders or symptoms not fitting AD were excluded. Donors with low RIN values (< 6.5) or additional findings of comorbid pathology were replaced. This resulted in 11 resilient donors which were matched with 12 AD patients and 12 control donors. A total of 35 donors were used for the experiments. For immunohistochemistry, donors without any available FFPE tissue were excluded, resulting in 7 resilient, 9 AD and 9 control donors

In total, 35 donors were selected with a full neuropathological assessment, available clinical data and a CDR or GDS score (Fig. 1). These included demented AD patients (CDR or GDS score of 3 or 7, respectively) with intermediate or high amounts of AD pathology (Braak 4–6, Thal ≥ 4), resilient donors with intact cognition (CDR 0–0.5 or Reisberg 1) [21, 22] and intermediate to high amounts of AD pathology (Braak 3–5, Thal ≥ 4) and cognitively intact age-matched controls (CDR 0–0.5 or Reisberg 1) with low amounts of AD pathology (Braak 1–2, Thal ≤ 2). Cases that showed any signs of psychiatric or neurological disease other than associated with AD were excluded from this study. In order to control for the amount of pathological comorbidities between our resilient and AD groups, we excluded AD patients with severe amounts of comorbid pathology (e.g. cortical LBs, LATE, HS). Donors were matched as closely as possible for sex, age, post-mortem interval, pH and ApoE genotype (Table 1).

**Table 1:** Summary of key demographics of matched groups

	Control	AD	Resilient	*P*
N	12	12	11	
Sex	6M/6F	6M/6F	5M/7F	0.895
Age	82.2 ± 10.6	82.2 ± 9.9	87.7 ± 8.2	0.274
pH	6.6 ± 0.3	6.5 ± 0.2	6.4 ± 0.2	0.393
PMI	6.0 ± 1.8	5.2 ± 1.0	6.4 ± 1.7	0.155
CDR/GDS	≤ 0.5/1–2	= 3/7	≤ 0.5/1–2	
ApoE-ε4	3+/9−	7+/5−	5+/6−	0.334
RIN	8.0 ± 0.8	8.1 ± 0.8	7.4 ± 0.6	0.066
BW	1162.8 ± 119.9	1088.7 ± 135.0	1196.4 ± 102.5	0.103
CERAD	0.1 ± 0.3	2.8 ± 0.6	1.5 ± 0.8	< 0.0001
Braak	1.3 ± 0.7	5.5 ± 0.8	4.3 ± 0.9	< 0.0001
Thal	0.7 ± 0.7	4.7 ± 0.8	3.5 ± 0.5	< 0.0001
TDP-43 (NC-LATE)	0	0	0.1	0.336
α-syn	0	0	0	
HS	0	1	0	0.358
Vascular pathology	0	2	1	0.326

*AD* Alzheimer’s disease, *α-syn* α-synuclein, *ApoE-ε4* Apolipoprotein E4 allele, *BW* brain weight (grams), *CERAD* Consortium to Establish a Registry for Alzheimer's Disease, *CDR* Clinical Dementia Rating, *F* female, *GDS* Global Deterioration Scale, *HS* hippocampal sclerosis, *M* male, *PMI* post-mortem interval (hours), *RIN* RNA integrity number, *TDP-43 (LATE-NC)* TAR DNA-binding protein 43, Limbic-predominant age-related TDP-43 encephalopathy neuropathologic changes

*P* = ANOVA-based *P-*value for continues variables and chi-square based *P*-values for categorical values. Data are represented as mean ± SD

### RNA isolations

Tissue from the SFG was used to make a tissue punch (Biopunch®, Ted Pella Inc., USA), 8 mm in diameter, containing all cortical layers to ensure that samples for RNA sequencing have a similar cellular content. Grey matter was dissected inside the cryostat with a pre-chilled scalpel and collected in pre-chilled tubes and kept on dry ice. Around 15 sections of 50 µm were cut, resulting in 15–20 mg of tissue that was used for RNA isolation with the RNeasy Mini Kit RNA (Qiagen, Valencia, CA, USA) in combination with Trizol (3 ml Trizol per 100 mg tissue; Life Technologies, Grand Island, NY, USA). After homogenization, phase separation was accomplished by addition of chloroform by vigorously shaking, incubating at room temperature for 2–3 min after which the samples were centrifuged for 15 min at 12000 g at 4 °C. The aqueous phase was mixed with an equal volume of 70% ethanol. Samples were then loaded on a RNAeasy Mini column (Qiagen, Valencia, CA, USA) and further processed according to manufacturer’s instructions. RNA yield and purity was determined using a Nanodrop. Quality of RNA was determined with the Agilent 2100 bioanalyzer (Agilent Technologies, Palo Alto, CA, USA) or 4200 Tapestation (Agilent Technologies). Only samples with RIN > 6.5 were included in the experiment (average RIN: 7.8, range 6.5–9.7; Table 1). For the isolation of small RNAs including miRNA and SNORDs, the same procedure was followed as described above after which small RNA molecules were isolated with the miRNeasy Micro Kit (Qiagen, Valencia, CA, USA) according to manufacturer’s instructions.

### Library preparation and read processing

NEBNext Ultra II Directional RNA Library Prep Kit for Illumina was used to process 35 samples (GenomeScan, Leiden, The Netherlands). Briefly, rRNA was depleted from total RNA using the rRNA depletion kit (NEB #E6310). After fragmentation of the depleted rRNA, a cDNA synthesis was performed for ligation with the sequencing adapters and PCR amplification. The size of the resulting products was consistent with the expected size distribution (a broad peak between 300 and 500 bp). Clustering and DNA sequencing using the NovaSeq6000 was performed according to manufacturer's protocols. A concentration of 1.1 nM of DNA was used. At least 15 Gb, yielding ~ 30 to 40 million read pairs, was generated per sample with a quality score of ≥ 30. Image analysis, base calling, and quality check was performed with the Illumina data analysis pipeline RTA3.4.4 and Bcl2fastq v2.20. Sequence reads were trimmed to remove possible adapter sequences using cutadapt v 2.10. Reads were aligned to the human genome (GRCh38.p13) using STAR (2.7.10a) with default settings. Feature counting was performed with HTSeq v0.11.0. Samples were sequenced in two batches, with a minimal batch effect between runs.

### Differentially expressed genes (DEG) analysis

Raw read counts were imported into R and normalized with DESeq2. Low abundant transcripts, normalized counts lower than or equal to 5 for at least 12 donors, were removed, resulting in 22,595 transcripts. Unwanted sources of variation, such as a possible batch effect between sequencing batches and sex were used as covariate. Principal component analysis (PCA) was performed using the plotPCA function [23] with varianceStabilizingTransformation (vst) genes, using the top 500 most variable genes after correcting for batch and sex. As sex was strongly associated with most of the variation in a PCA, we investigated sex-specific gene expression by combining sex and group into a single factor with all combinations of the original factors. Another separate analysis to further investigate sex-related differences was performed in which genes belonging to the X and Y chromosomes were excluded. DEGs between control, AD and resilient donors were determined using DESeq2 (version 1.40.2) (adjusted p ≤ 0.1) and apelgm (version 1.22.1) for calculating fold changes [24]. To investigate the effects of pathology, genes of interests were correlated (spearman correlations) with the local quantified pathology, based on the IHC signal from AT8 for pTau and 4G8 for plaques and a separate DEGs analysis was performed in which the local quantified pathology was used as a covariate.

### Cell type proportions

The proportion of different cell types was estimated using cell type deconvolution with dtangle [25] by using Sutton et al. [26] as a template together with sets of marker genes from Hodge et al. [27] and Mathys et al. [28]. Cell-type markers were selected as the top 1% of markers using its find_markers() function with method = ”diff”.

### GSEA and pathway analysis

To investigate if there are sets of genes enriched in the different groups, we performed preranked gene sets enrichment analysis (GSEA) with the R package fgsea [29] (version 1.26.0) using the gene sets from the Molecular Signatures Database (MSigDB, version 7.5.1), including canonical pathways (C2, consisting of Reactome, WikiPathways, BioCarta, KEGG and PID gene-sets) and GO (C5), with 1000 permutations. Genes were ranked based on the Wald statistic from DESeq2.

### Weighted correlation network analysis (WGCNA)

To identify gene modules associated with resilience or AD pathology, we used the top 50% most variable genes from the vst genes from DESeq2, corrected for batch and sex, as input for the R implementation of WGCNA [30]. Hierarchical clustering by distance was used to detect outlier samples, which resulted in the exclusion of 1 control donor. WGCNA clustering was performed using the “1-TOMsimilarityFromExpr” function, network type “signed”. Soft threshold power was 14, as determined by a scale-free topology power of 0.90 and mean and median connectivity around 100. DeepSplit was 2 and a minimum module size of 30 was used, resulting in 26 modules. Correlations between module eigengenes, which represent the first principle component of the gene expression within each module, and neuropathology were done using Pearson correlations and corrected for multiple testing with Benjamin-Hoch. Differences between module eigengenes and groups were done with t-tests and directions were visualized using the differences between means. Hub genes were selected based on gene connectivity and by using the MMC algorithm of CytoHubba. Overrepresentation analysis on the modules identified by WGCNA was performed with the R package clusterProfiler [31] using gene ontology (GO) (GO.db version 3.17.0) and ReactomePA [32] using reactome pathways (reactome.db version 1.84.0). For the overrepresentation analysis, the genes belonging to each module were tested against all genes used in the WGCNA analysis. Enrichment of genes belonging to the different modules in the different groups was tested by using the modules as gene sets as input for GSEA using fgsea. Relative VST-transformed counts across samples (VST gene–gene averages across all samples) were used for heatmaps to visualize expression of genes belonging to their modules.

### Quantitative PCR

For cDNA synthesis of mRNA, 250 ng of mRNA for each donor was transcribed to cDNA using the QuantiTect Reverse Transcription Kit (Qiagen), after which cDNA was stored at − 20 °C. For the cDNA synthesis from miRNA, either the miRCURY LNA miRNA Kit (Qiagen) was used using 100 ng of miRNA as input or the TaqMan MicroRNA Reverse Transcription Kit (Thermofisher) with 10 ng input of miRNA and with probes for u6 and SNORD114-6. qPCR was performed using the Sybr Green mastermix (Qiagen) and SNORD114-6 primers (forward primer: TGGACTAATGATGTCCACTGGT, reverse primer: TGGACCTCAGAGTTCCAGACATATATTC) or using TaqMan fast advanced master mix (Thermofisher). The mean expression of U6 miRNA or EF1a and GAPDH for mRNA were used for normalization. Ct values were determined using the second derivative method after which fold changes were calculated using the ΔΔ Ct method.

### Immunohistochemistry

Cryostat sections (10 µM), adjacent to the sections used for RNA isolations, were used to determine the regional pathological load of all donors. Formalin-fixed paraffin embedded (FFPE) blocks from medial frontal gyrus, SFG or inferior frontal gyrus from a sub selection of the donors (Fig. 1) were acquired from the NBB. 8 µm sections were cut for IHC validation of targets derived from the bioinformatics analyses.

Briefly, cryostat sections were post-fixated for 10 min in 4% PFA in PBS. For the Aβ staining, sections were boiled in sodium citrate buffer (0.01 M citrate buffer, 0.05% tween-20, pH 6.0) at 700W in a microwave and incubated with 70% formic acid for 10 min. Sections were blocked with 5% milk (ELK, the Netherlands) for 30 min at room temperature (RT). For the pTau staining, sections were incubated with primary antibody after post-fixation. FFPE sections were deparaffinized in xylene and rehydrated in a graded ethanol series. Sections were boiled in sodium citrate buffer for 10 min at 700W in a microwave. Both cryostat and FFPE sections were blocked with 5% fetal calf serum for 30 min at RT. Primary antibodies were incubated overnight at 4 °C (anti-p-Tau AT8, Thermo, USA, 1:2000; anti-amyloid 4G8, Signet, MA, USA, 1:10,000; MT-CO1, Abcam, United States, 1:200; MT-I/II, Abcam, United States, 1:500; phosphorylated PERK (pPERK), Santa Cruz, 1:6000; Heat shock protein 70 (HSP70), Santa Cruz, 1:200, X-box binding protein 1, spliced (XBP1s), Cell Signaling, 1:200). Horse anti-mouse-HRP (DAKO, Denmark; 1:400) or HRP secondary antibody from EnVision Detection Systems (K8023; DAKO, United States) were incubated as secondary antibodies for 1 h at RT. Sections treated with Horse anti-mouse-HRP were also incubated with ABC (Vector Labs, USA). All sections were developed with DAB (K8023; DAKO, United States). For double and triple immunofluorescent IHC, FFPE sections were were deparaffinized in xylene, rehydrated in a graded ethanol series and boiled in sodium citrate buffer at 700W in a microwave for 10 min. Sections were blocked with 5% fetal calf serum for 30 min RT. Primary antibodies were incubated overnight at 4 °C (MT-CO1, Abcam, United States, 1:100; MT-I/II, Abcam, United States, 1:250; pPERK, Santa Cruz, 1:3000; XBP1s, Cell Signaling, 1:100; HSP70, Santa Cruz, 1:100; NeuN, Millipore. 1:500; Glial fibrillary acidic protein (GFAP), DAKO, 1:250; GFAP-Cy3 conjugated, Sigma-Aldrich, 1:250; Ionized calcium-binding adapter molecule 1 (Iba1), DAKO, 1:250) and secondary antibodies (donkey anti-mouse Cy3,ThermoFisher Scientific; donkey anti-mouse Alexa Fluor 488, ThermoFisher Scientific; donkey anti-rabbit Cy3, ThermoFisher Scientific; donkey anti-rabbit Cy5, ThermoFisher Scientific; goat anti-mouse IgG1 Alexa Fluor 488, ThermoFisher Scientific; goat anti-mouse IgG2A Alexa Fluor 488, ThermoFisher Scientific; goat anti-mouse IgG1 Cy3, ThermoFisher Scientific; goat anti-mouse IgG2A Alexa Fluor 647, ThermoFisher Scientific, all 1:400) were incubated for 1 h at RT. Sections were counterstained with DAPI and autofluorescence was quenched with 0.1% sudan black for 5 min.

Pictures were taken with a slide scanner (Axio slide scanner, ×20 magnification) or with a Leica SP5 confocal microscope (63× magnification, resolution of 1024 × 1024 dpi and 100 Hz speed). Two regions of interest (ROI) were selected per section, containing all cortical layers. Markers were quantified based on the optical density (OD) as described previously [33]. In brief, threshold was set to three times the background (OD) for all experiments, except IHC for metallothionein, in which a threshold of to 1.5 times was used. Within the outlined area, signal that was higher than the threshold was considered the positive surface area. The integrated optical density (IOD) was calculated by multiplying the positive surface area with the OD which was divided by the total area of the ROI to obtain the corrected IOD (cIOD). For the double and triple immunofluorescent IHC, representative pictures were taken from the gray matter.

### In situ hybridization

The sequence for the LNA oligoribonucleotide probe for SNORD114-6 was: 5′-FAM-AUGAUUUATACGCCACCAGUGGACA-3′ with FAM denoting a fluorescein tag and a locked nucleic acid (LNA) after the FAM, the 9th position and at the 3′ end (Eurogentec).

In situ hybridization was performed as previously described [34]. In brief, FFPE sections were de-paraffinized and boiled in sodium citrate buffer (pH 6) for 10 min in a microwave. After de-proteinization with proteinase K at 37 °C for 15 min and de-lipidation with PBS/triton, sections were prehybridized in hybridization mix for 2 h at RT. Hybridization was performed at 55 °C overnight and subsequently washed in 5× SCC, 2× SCC, 0.2 SCC (at 55 °C) and in PBS (at RT). To detect the LNA probe, sections were incubated with rabbit anti-fluorescein-alkine phosphatase for 3 h at RT. Signal was developed with NBT/BCIP color substrate and stopped with distilled water, and sections were washed with methanol.

### Statistical analysis

Data collected from IHC were tested for normality by the Shapiro–Wilk test, followed by either ANOVA with Tukey’s multiple comparisons test or Kruskal–Wallis with Dunn’s multiple comparisons test. Statistical analyses were performed using RStudio (2023.06.1) for R (4.3.1). P-values of < 0.05 were considered significant.

## Results

### Donor demographics

To identify individuals who might be resilient to AD, donors with a discrepancy between their cognition and the amounts of AD neuropathology were selected from the brain collection of the NBB. Donors were carefully selected based on their pathological and clinical information (Fig. 1) and classified into three different groups: Control donors with low amount of AD pathology and no cognitive impairment, AD patients with intermediate to high amounts of AD pathology and cognitive impairment, and resilient individuals with intermediate to high amounts of AD pathology and no cognitive impairment. Key donor demographics were well matched across the three different groups including age, post-mortem interval, pH and sex (Table 1).

**Fig. 2:**
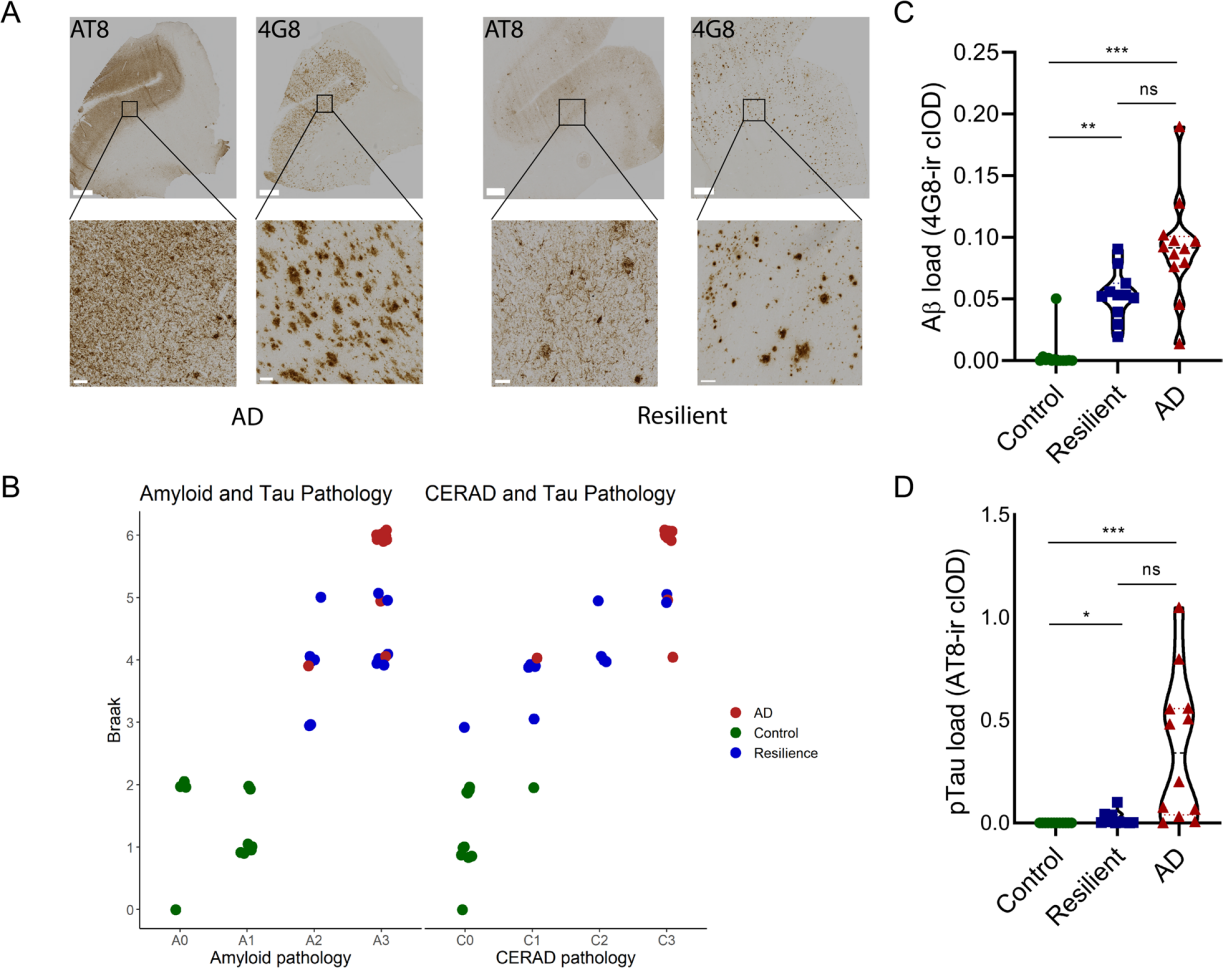
Neuropathological characterization of the cohort used for the RNA sequencing and immunohistochemical experiments. 35 samples were included and categorized based on the amount of AD neuropathology and cognition. **A** Examples of section of the SFG of donors with intermediate to high amount of pTau (AT8) and beta amyloid (4G8) pathology that were included in the AD (left panels) and resilient group (right panels). **B** Neuropathological scores of the different groups with amyloid pathology on the x-axis (Thal and CERAD according to the ABC score from the National Institute on Aging-Alzheimer’s Association guidelines) and tau pathology (Braak stage). **C** The plaque load was significantly higher in both resilient and AD compared to control (Kruskall–Wallis; H = 25.63, p < 0.0001, AD vs control; p =  < 0.0001, resilient vs control; p = 0.005, resilient vs. AD; p = 0.252). **D** Quantification of the pTau load, showing significantly more pTau in the resilient and AD groups (Fig. 2D: Kruskal–Wallis H = 23.08, p < 0.0001, AD vs control; p =  < 0.0001, resilient vs control; p = 0.037, resilient vs AD; p = 0.085). Of note, there is a trend of increased pTau pathology in the AD group compared to the resilient group. Scale bars in panel A indicate 1 mm in upper panel and 50 µm in lower panel. p < 0.05: *, p < 0.01: **, p < 0.001: ***. ns = not significant

In both the AD and resilient groups significant amounts of Aβ plaques and pTau was present in the SFG (Fig. 2A). Likewise, global AD pathology based on the Braak and Thal scores was higher in AD and resilient groups than in the control group (Fig. 2B; Table 1, Braak: Kruskal–Wallis H = 27.96, p < 0.0001, AD versus control; p < 0.0001, resilient versus control; p < 0.01, resilient versus AD; p = 0.159, Thal: Kruskal–Wallis H = 28.47, p < 0.0001, AD versus control; p < 0.0001, resilient versus control; p < 0.001, resilient versus AD; p = 0.210). Likewise, the amount of neuritic plaques, based on CERAD, were also significantly higher in the AD and resilient group compared to control. Average CERAD scores were higher in AD compared to resilient, albeit not significant (Fig. 2B; Table 1 CERAD: Kruskal–Wallis H = 26.99, p < 0.0001, AD versus control; p < 0.0001, resilient versus control; p < 0.002, resilient versus AD; p = 0.227). Whereas AD patients were demented (GDS = 7 or CDR = 3), both resilient and control groups were cognitively intact (GDS ≤ 2 or CDR ≤ 0.5) (Table 1). More specific quantification of both the amount of plaques measured by 4G8 and pTau measured by AT8 in sections of the GFS adjacent to the tissue used for the RNA sequencing of each donor demonstrated in both resilient and AD significantly more amyloid plaque pathology compared to control (Kruskall–Wallis; H = 25.63, p < 0.0001, AD vs control; p ≤ 0.0001, resilient vs control; p = 0.005) and pTau (Fig. 2D: Kruskal–Wallis H = 23.08, p < 0.0001, AD vs control; p ≤ 0.0001, resilient vs control; p = 0.037). A trend towards more pTau pathology in AD compared to the resilient group was present (p = 0.085). There was also a trend towards more ApoE4 genotypes in the AD group compared to the control group (p = 0.097), which is representative of this population [35]. ApoE genotypes between the resilient and AD groups were well matched. In addition, we found a striking difference between the amount of comorbid pathology, such as LBs, TDP-43 or vascular pathology, present in our original resilient samples and all AD samples that matched our inclusion criteria (Fig. 1). As these comorbidities were often absent in our resilient donors and can also contribute to cognitive decline, we matched for comorbid pathology. Consequently, there was no significant enrichment of pathological comorbidities in any of the groups (Table 1). As non-significant differences in the amount of AD pathology between the resilient and AD group may possibly influence our main outcomes, we controlled also for the amount of AD pathology in separate analyses.

### Transcriptional differences in the SFG between controls, resilient and AD donors

**Fig. 3:**
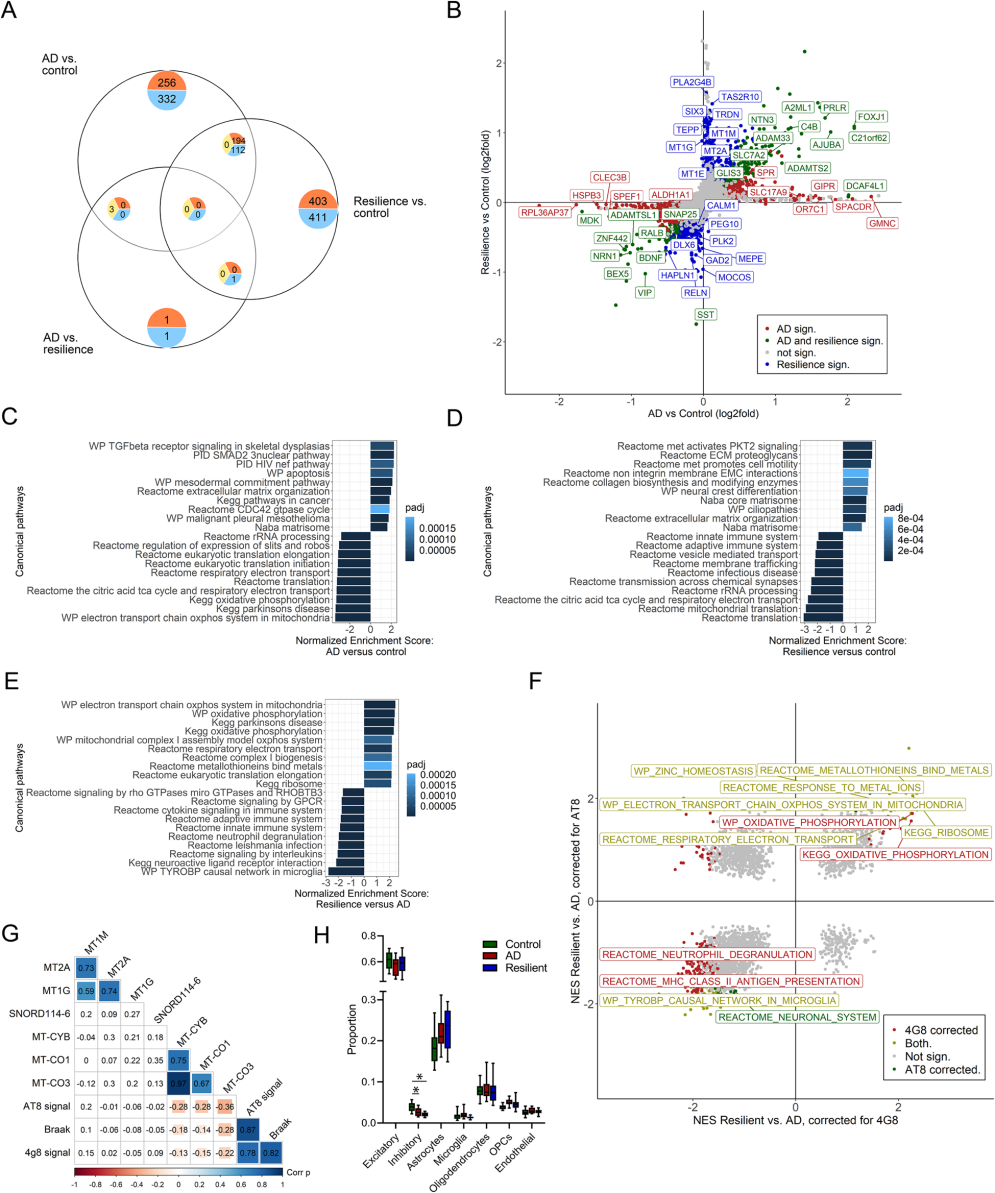
Transcriptional differences between AD and resilient donors become apparent at the gene-set level. **A** Venn diagram of DEGs: 897 between AD and control, 1121 between resilient and control and 6 between AD and resilient. Red are upregulated genes, blue downregulated genes. **B** Quadrant plot of fold changes between resilient versus control and AD versus control. Unique DEGs between resilient and control are highlighted in blue, between AD and control in red and between both resilient and AD versus control in green. **C**–**E** Top GSEA results between the different groups. Enriched gene sets in AD include processes related to the extracellular matrix, apoptosis and immune function, while in controls processes related to mitochondrial processes and translation are enriched. Enriched gene sets in the resilient group are related to the extracellular matrix and ciliopathies. Compared to AD, **E** Top GSEA enriched processes in the resilient group include changes in mitochondria and in metabolism of heavy metals. **F** Quadrant plot of GSEA of resilient versus AD corrected for pTau or Aβ. When corrected for either pTau or Aβ, processes related to metallothionein are enriched in the resilient group. Correcting for pTau partly removes significance of mitochondrial processes and immune functions. **G** Correlation matrix of top DEGs or enriched genes between resilient and AD and AD pathology. Mitochondrial genes negatively correlate with pTau pathology, while metallothionein genes and SNORD114-6 do not correlate with AD pathology. Red is negatively and blue positively correlated. Colored boxes indicate significance. **H** Estimated cell type proportion shows a lower proportion of inhibitory cells in the resilient and AD groups compared to the control group. Statistical significance was tested with a two-sided Student’s t test. p < 0.05: *

To gain insight in possible mechanism which could help to explain how resilient individuals maintain cognition despite the presence of AD pathology, we performed bulk RNA-sequencing on RNA isolated from the grey matter of the SFG of resilient, AD and control donors. 897 genes were significantly different between AD and control, 1121 between resilient and control (Fig. 3A; Additional file 1). Most DEGs in the comparisons of AD versus control and resilient versus control had a similar direction (Fig. 3B). Notably, growth factors such as brain derived neurotrophic factor (BDNF) and neuritin (NRN1), or genes previously associated with cognition in relation to AD [81], such as plexin B1 (PLXNB1), were both downregulated in resilience and AD compared to control. Likewise, markers related to interneurons such as vasoactive intestinal peptide (VIP) and somatostatin (SST) were downregulated in both AD and resilient or only resilient donors, respectively (Fig. 3B; Additional file 1). Remarkably, when comparing gene expression between resilient and AD donors, only 6 genes were differentially expressed. Despite the few DEGs between the resilient and AD groups, they indicate possible relevant changes between these groups, such as changes in mitochondrial genes (*MT-CO3*), changes in apoptosis and Aβ production (*PAR-4*) or the involvement of snoRNA’s (*SNORD114-6)* (Additional file 1). In addition, in a recent snRNA-seq. study, 5 DEGs were found in excitatory neurons between resilient and AD cases. In the current dataset these genes are also lower expressed in the majority of the AD patients compared to the control and resilient donors, albeit not significant (Additional file 2). These observations suggest that statistically significant single gene changes in the SFG associated with resilience versus AD are subtle. This led us to hypothesize that differences in gene expression between AD and resilient individuals may become apparent when analyzing changes at the gene-set level.

### Gene sets related to mitochondria and glial cells are more highly expressed in resilience

In order to get insight in which biological processes might be differentially regulated between the groups, we performed GSEA [36]. When comparing the AD and resilient groups to the control group, gene sets involved in processes related to the extracellular matrix were more highly expressed, whereas gene sets related to mitochondria where more highly expressed in the control group (Fig. 3C, D; Additional file 1). Interestingly, when comparing resilient to AD donors, gene sets involved in processes related to mitochondrial functioning were more highly expressed in the resilient group (driven by, amongst others, *MT-CO1, MT-CO3, MT-CYB*) (Fig. 3E), of which *MT-CO3* was also a DEG in the resilient compared to the AD group. In addition, gene sets related to the response to heavy metals and metallothionein (MT) signaling (driven by *MT1G, MT2A, MT1M*) were more highly expressed in resilient compared to AD, while genes related the innate and adaptive immune system and TYRO protein tyrosine kinase-binding protein (*TYROBP*) signaling were more highly expressed in AD compared to resilience. A complete list of the top DEGs and GSEA results is provided in Additional file 1.

### Effects of cell-type proportion

Next, we investigated if there are any changes in the cell-types by estimating the cell-type composition from the bulk gene expression data using single nucleus RNA-seq. datasets as a general reference [27, 28]. When comparing resilient to AD, there were no significant differences in the estimated proportions of astrocytes and microglia. Thus, altered expression of genes involved in mitochondrial-, and immune processes, and metallothionein and *TYROBP* function is not driven by changes in cell-type proportions, but likely due to different cellular responses to AD pathology. Recently, higher proportion of interneurons and interneuron subtypes were found in resilient donors compared to AD patients [28]. However, in our dataset there was a lower proportion of inhibitory cells in both the resilient and AD cases compared to the control cases (Fig. 3H).

### Effects of pathology on main outcomes

To further validate if the changes in gene expression are resilient-specific rather than due to a difference in Aβ and pTau load, a separate DE analysis was performed using the quantified amounts of pathology as covariate. When controlling separately for the amounts of pTau (AT8) or plaque levels (4G8), gene sets encoding for MT signaling are still more highly expressed in the resilient group compared to the AD group (Fig. 3F, top right quadrant). However, mitochondria or immune related processes were only more highly expressed in the resilient group or AD group, respectively, when controlling only for the amounts of plaques. This was not the case when only controlling for the amount of pTau, which partly removed significance. Likewise, expression levels from our initial analyses of DEGs related to mitochondrial processes negatively correlated with pTau levels while this was not the case for the MT genes (Fig. 3G; Additional file 1). This suggests that MT signaling could be involved in resilience even when pathology further progresses, while increased expression of mitochondrial related genes could be an initial compensatory response, which diminishes with progression of pathology.

### Co-expression networks in resilience

**Fig. 4:**
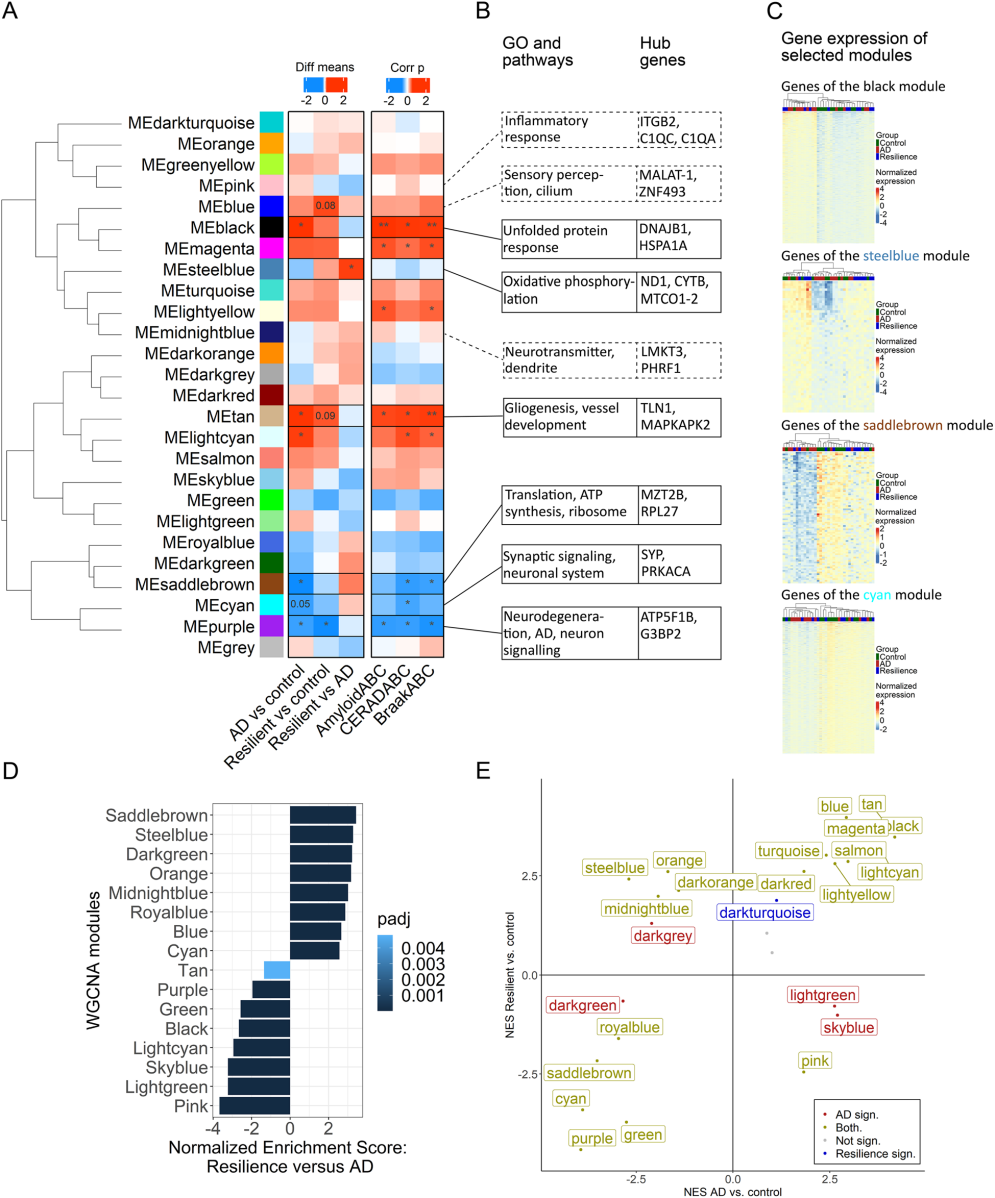
WGCNA identifies modules that are differentially associated with pathology and the AD and resilient groups. **A** Module-trait correlation of the 26 modules identified in relation to group and AD pathology with their relatedness. Notably, some modules correlate with pathology (ABC score for Thal, Braak and CERAD stages) and were significantly different between the different groups (such as the black, tan or saddlebrown modules), which were chosen as modules of interest. Significant module correlations with pathology and significant differences between groups are highlighted with p < 0.05: *, p < 0.01: ** and p < 0.001: ***. Trends between the different groups are indicated with p-values. **B** Results of the gene ontology (GO) and pathway analyses related to the genes in each module and the hub genes which could be regulators of these modules. GO and pathways of modules which correlate significantly with resilient or control are in boxes with solid lines and other relevant modules with dashed lines. **C** Heatmap of relative VST-transformed counts across samples of the genes in selected modules of interest. Note that donors cluster as group based on gene-expression in these selected modules. **D** Top GSEA results between the resilient and AD groups using the modules identified by WGCNA as input. Modules significantly enriched (padj < 0.05) are plotted with normalized enrichment score. **E** Quadrant plot of normalized enrichment score (NES) of modules in bulk RNA-seq. gene expression using GSEA. Both the resilient group and AD group are compared and plotted against controls. Modules significantly enriched (padj < 0.05) in resilient versus control are depicted in blue, AD versus control in red and in both groups versus control in yellow. The pink, midnight blue, steelblue and skyblue show an opposite enrichment, highlighting additional biologically relevant modules

To determine if there are biological relevant sets of genes that are expressed together, a weighted gene co-expression network analysis (WGCNA) was performed [30]. WGCNA resulted in a list of 26 gene modules (Fig. 4A). Interestingly, while several modules correlated with both amyloid and tau pathology, they were significantly different between the resilient and AD groups or between the AD and control groups (e.g. note the lightcyan, tan, saddlebrown and black modules, Fig. 4A). In addition, there were also modules that were significant between the resilient and AD groups (such as the steelblue module). This suggests that the genes belonging to these modules may play a role in resilience towards AD pathology. These modules of interest were further investigated using gene ontology (GO), pathway analysis and by identifying hub genes, which often play a central role in the modules.

The modules with the strongest correlations with AD pathology and significant differences between the groups included the black, tan and saddlebrown modules. The black module was related to heat shock proteins (HSPs) and the unfolded protein response (UPR), was positively correlated with pathology and its eigengenes were significantly higher in the AD group compared to the control group. This was also substantiated by the expression of genes belonging to the black module, in which most AD donors had increased expression of these genes (Fig. 4C). This suggests that HSPs and the UPR are activated more in the AD group as a reaction to AD pathology, while this reaction is lower in the resilient group. The module saddlebrown contained genes related to translation, ribosome function and ATP synthesis. This module was significantly different between the AD and control group, which was also substantiated by the gene expression in this module, as in most AD donors the genes belonging to this module were downregulated (Fig. 4C). Lastly, the tan module, related to gliogenesis and vessel development, was positively correlated with AD pathology and its eigengenes were significantly higher in the AD compared to the control. Taken together, the identification of these modules by WGCNA suggest a different reaction towards AD pathology in the resilient and AD groups. In particular, cellular homeostasis might be better maintained in the resilient donors reflected by differential expression of module of genes involved in processes such as translation, energy metabolism and the UPR.

Other modules of interests include the cyan module and steelblue module. The cyan module, related to synaptic signaling, showed a trend towards being lower expressed in the AD group compared to the control group The expression of genes belonging to this module showed inter-donor variability, although most of the AD donors had a reduced expression of these genes (Fig. 4C). Interestingly, one of the hub genes, synaptophysin, often used as a marker for synapses, was increased in resilient donors, as was also shown by others [5, 6, 37, 38]. The steelblue module was significantly different between the resilient and AD group, and contained many genes related to mitochondrial processes. Genes from the steelblue module were also downregulated in AD donors compared to the other groups (Fig. 4C). In particular, many mitochondrial derived RNAs belong to this module, which could indicate a higher activity or higher number of mitochondria in the resilient compared to the AD donors. The genes belonging to this module show overlap with the DEGs between the resilient and AD group and the gene sets related to mitochondrial processes that where more highly expressed in the resilient compared to the AD groups. Of note, the blue module, of which the eigengenes showed a trend towards being higher in the resilient donors compared to the control donors, contained the MT-I/II genes. These were also more highly expressed in the resilient compared to the AD donors in the initial GSEA analysis.

To further explore the different modules might be differentially regulated between the different groups, GSEA was performed using the genes of each module as a gene set. This allowed to see if the genes belonging to a specific module were more highly expressed in one of the groups. This confirmed that the genes belonging to the black, tan, saddlebrown or steelblue modules were more highly expressed in the resilient or the AD group (Fig. 4D). Notably, genes from other modules were also more highly expressed in the resilient or AD group. Moreover, some of these modules contained genes with an opposite enrichment in the resilient or AD group when compared to the control group (Fig. 4E, top left and bottom right quadrant). For example, genes belonging to the pink or midnightblue module were more highly expressed in the AD or resilient group, respectively. The pink module is related to inflammatory response and adaptive immune responses and contains genes such as triggering receptor expressed on myeloid cells 2 (TREM2), TYROBP, tyrosine-protein kinase (SYK). The hub genes belonging to the pink module were, amongst others, complement genes (Fig. 4B; Additional file 3). This is in line with the GSEA results in which the genes related to the TYROBP pathway were more highly expressed in the AD compared to the resilient group. The midnightblue module is related to neurotransmitter activity and dendrites, and contains, amongst others, glutamate ionotropic receptor NMDA type subunit 1 (GRIN1), diacylglycerol lipase alpha (DAGLA) and syntaxin 4 (STX4) (Additional File 3). This suggests that genes related to dendrites are differentially regulated in AD and resilience. Furthermore, the skyblue module, which was more highly expressed in the AD group, was related to the unfolded protein response. This module, in addition to the black module, suggests that the unfolded protein response is increased in the AD donors compared to the resilient individuals. Of note, genes from other modules with a different enrichment in AD and resilience compared to control (Fig. 4E) were not linked to known biological functions, based on GO and pathway analysis (Additional File 3).

### Immunohistochemical validation of metallothionein, mitochondrial proteins and the unfolded protein response

**Fig. 5:**
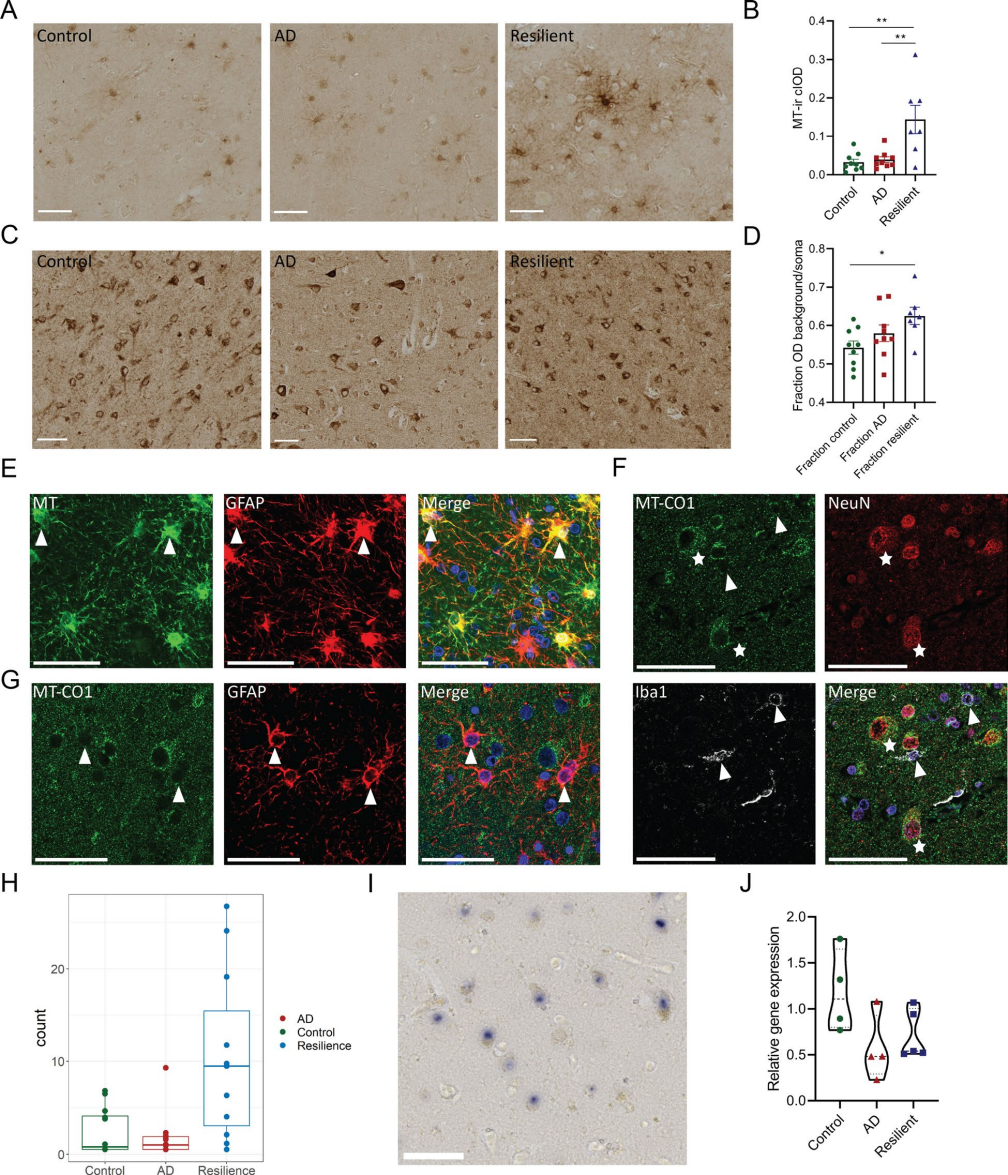
Validation of metallothionein, mitochondrial changes and SNORD114-6 with IHC or in situ. **A** Representative images of metallothionein levels the control, AD and resilient groups. **B** Quantification of metallothionein immunoreactivity (MT-ir). MT-ir is significantly increased in resilient compared to control cases (**A**–**B**: F(2,22) = 5.29, p = 0.0008, AD versus control; p = 0.964, resilient versus control; p = 0.001, resilient versus AD; p = 0.003) **C** Representative images of mitochondria using MT-CO1. **D** Quantification of the proportion of OD from MT-CO1 in and outside of the soma. The proportion of MT-CO1 outside compared to inside of the soma is significantly larger in the resilient compared to the control cases (F(2,22) = 3.87 p = 0.036, AD versus control; p = 0.387, resilient versus control; p = 0.028, resilient versus AD; p = 0.299). **E** Fluorescent IHC stains show that metallothionein (green) is present in GFAP-positive astrocytes (red). Double-labeled astrocytes are denoted with an arrow, nuclei are stained with DAPI (blue). **F**–**G** MTCO1 (green) is primarily present in NeuN-positive neurons (red) and less in Iba1-positive microglia (white) or GFAP-positive astrocytes (red). Double-labeled astrocytes are denoted with an arrow and neurons with an asterisk, nuclei are stained with DAPI (blue). **H** Normalized counts of SNORD114-6 in the different groups. **I** In situ signal of SNORD114-6 in the nucleolus in neurons. **J** Relative gene expression of SNORD114-6 with qPCR. There are no statistical differences between the groups. Scale bars in all panels are 50 µm. Data of IHC is represented as average ± SEM, in situ as relative gene expression to housekeeping genes. p < 0.05: *, p < 0.01: **

**Fig. 6:**
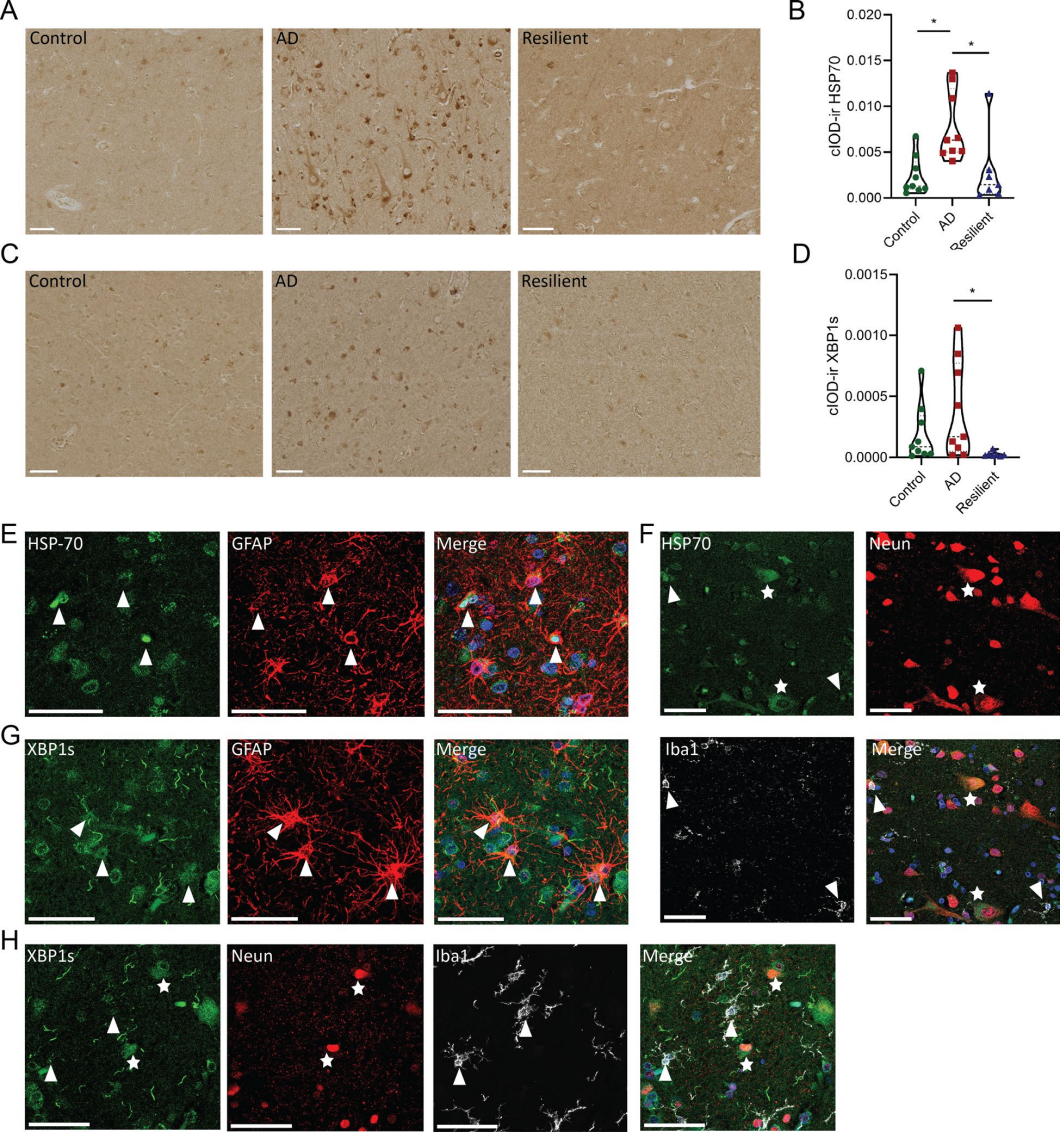
Validation of the unfolded protein response with IHC. **A** Representative images of HSP70 levels in the different groups. **B** Quantification of HSP70 immunoreactivity (HSP70-ir), which is significantly increased in the AD compared to the resilient and control cases (Kruskal–Wallis H = 10.81, p < 0.0045, AD versus control; p = 0.0145, resilient versus control; p > 0.999, resilient versus AD; p = 0.0146). **C** Representative images of XBP1s in the different groups. **D** Quantification of XBP1s immunoreactivity (XBP1s-ir), which is significantly increased in the AD compared to the resilient cases (Kruskal–Wallis H = 8.73, p < 0.0127, AD versus control; p > 0.999, resilient versus control; p = 0.0971; resilient versus AD; p = 0.0116). **E** and **F** Fluorescent IHC stains show that HSP70 (green) is present in GFAP-positive astrocytes (red), NeuN-positive neurons (red) and in Iba1-postive astrocytes (white). Double-labeled astrocytes are denoted with an arrow and neurons with an asterisk, nuclei are stained with DAPI (blue). **G** and **H** Fluorescent IHC images show XBP1s (green) is present in NeuN-positive neurons (red), GFAP-positive astrocytes (red) and Iba1-positive microglia (white). Double-labeled astrocytes are denoted with an arrow and neurons with an asterisk, nuclei are stained with DAPI (blue). Scale bars in all panels are 50 µm, p < 0.05: *

We observed changes in genes involved in detoxification of heavy metals (encoding for MT-I/II), genes involved in mitochondrial function and genes related to HSPs and the UPR in the resilient compared to the AD donors in the GSEA, even after adjusting for AD pathology, or in the WGCNA analysis. To gain more insight into the biological relevance of these changes we studied the protein expression of MT-I/II, MT-CO1, HSPA1A, XBP1s and pPERK, using IHC in the frontal cortex on a subset of the selected donors (Fig. 1). In the resilient group, but not in the AD group, MT-I/II staining was significantly higher compared to the control group (Fig. 5A, B: F(2,22) = 5.29, p = 0.0008, resilient versus control; p = 0.001, resilient versus AD; p = 0.003). MT-I/II was expressed in astrocyte-like cells as shown by the co-staining with GFAP (Fig. 5E), which confirms previous observations [39]. These data suggest that astrocytes in resilient donors have higher detoxification of heavy metals. Using the marker MT-CO1, which belongs to many of the significantly more highly expressed gene sets related to mitochondria in the resilient compared to the AD group, and an important gene in the midnightblue module, a higher proportion of MT-CO1 signal outside of the soma versus the soma itself was observed in resilient compared to control donors (Fig 5C, D: F(2,22) = 3.87 p = 0.036, resilient versus control; p = 0.028). Staining for MT-COI was found primarily in neurons (Fig. 5F, G), but was also present in astrocytes and Iba1-positive microglia. This suggests that mitochondria outside the cell soma express more MT-CO1 or that there are more mitochondria present outside the soma in for example dendrites or synapses in resilient individuals compared to the control donors. Finally, IHC was performed for one of the hub genes of the black WGCNA module (HSPA1A, or HSP70) and a relevant gene of the UPR pathway with high intramodular connectivity (XBP1). Higher levels of HSP70 and XBP1s were found in the AD group compared to the control and resilient groups (Fig. 6A, B: Kruskal–Wallis H = 10.81, p < 0.0045, AD versus control; p = 0.0145, resilient versus control; p > 0.999, resilient versus AD; p = 0.0146) or only to the resilient group, respectively (Fig. 6C, D: Kruskal–Wallis H = 8.73, p < 0.0127, resilient versus AD; p = 0.0116). Both markers were found in in glial cells but were primarily found in neurons (Fig. 6E–H). UPR activation is activated via three distinct signaling pathways, including the activating transcription factor 6 (ATF6), inositol-requiring enzyme 1 (IRE1)-XBP1 and PERK-eukaryotic Initiation Factor 2 alpha (eIF2α) pathways [40]. As the latter two pathways have been shown to be activated in AD patients [41], the marker pPERK was also used to investigate for possible differences in both cascades. However, no significant differences were found between the groups (Additional file 4). Based on these results, it is likely that HSPs and the IRE-XPB1s cascade of the UPR are more activated in the AD patients compared to the control and resilient donors.

### SNORD114-6

Interestingly, the gene with the highest fold change between the resilient and AD group was a non-coding RNA, nucleolar RNA, C/D Box 114-6 (SNORD114-6). By using ribosomal depletion in our library preparation, we were able to efficiently detect coding as well as non-coding transcripts. As the normalized counts were relatively low (Fig. 5H), we further attempted to validate SNORD114-6 using qPCR and in situ. SNORD114-6 is a ncRNA found in the nucleolus, which is in concordance with the results of the in situ hybridization (Fig. 5I). However, using the original isolated mRNA, we were not able to detect SNORD114-6 with qPCR. Using miRNA isolated from the same tissue blocks, we were able to pick up this ncRNA using SYBR green, while using TaqMan SNORD114-6 was undetectable. Nevertheless, no differences were found between the groups using SYBR green (Fig. 5J). Thus, in the current study it was impossible to further validate the increased levels of SNORD114-6 in the resilient donors.

### Sex differences in resilience

**Fig. 7:**
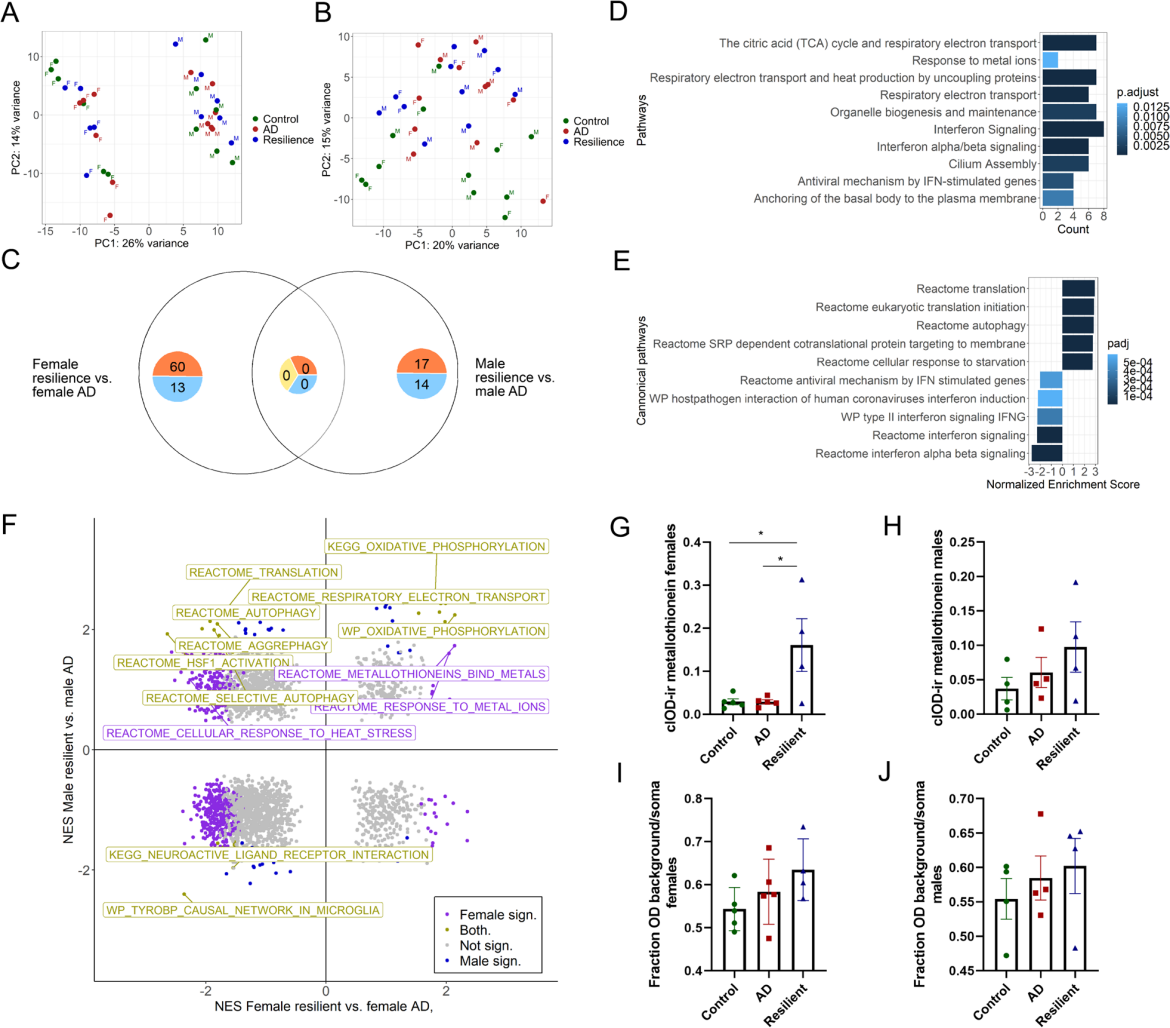
Sex-specific resilient mechanisms are related to autophagy and interferon signaling. **A** PCA plot of our data without correcting for sex shows PC1 is driven by sex. m = males, f = females. **B** When removing all genes from the X and Y chromosomes, sex is no longer driving PC1 and PC2 in the PCA plot. m = males, f = females. **C** Venndiagram showing the DEGs specific for female resilience or males resilience, with females having more DEGs. **D** Pathways which are overrepresented in the female DEGs are related to metallothionein (response to metal ions), mitochondria and interferon signaling. **E** GSEA of male versus female resilient donors after removing the X and Y chromosomes, indicating enrichment of gene sets related to autophagy and translation in males and interferon signaling in females. **F** Quadrant plot of normalized enrichment scores (NES) after performing GSEA on sex-specific gene-expression to explore sex-dependent mechanisms. Our main findings seem to be independent of sex. Metallothionein signaling, oxidative phosphorylation and the TYROBP pathway are all either up or downregulated in resilient compared to AD. Processes related to autophagy and translation are enriched in male resilient donors but not in females. **G**–**J** Quantification of MT-1/MT-II and MT-CO1 on protein level show that both pathways behave similarly in each sex, although only metallothionein signaling is significantly increased in female resilient cases compared to female AD cases (F(3, 14) = 5.86 p = 0.018, AD versus control; p > 0.999, resilient versus control; p = 0.029, resilient versus AD; p = 0.029). Data of IHC is represented as average ± SEM

Notably, one of the major drivers in the PCA, without correcting for any covariates, was sex. (Fig. 7A). This effect was largely negated after running a separate analysis in which genes belonging to the X and Y chromosomes were removed (Fig. 7B). As sex plays an important role in the pathophysiology of AD [42] and possibly also in resilience [43], we investigated if there are sex-dependent gene expression changes associated with resilience. When looking at sex-specific gene expression changes between resilient and AD donors, there were 72 DEGs between the female resilient and female AD donors and 32 between the male resilient and male AD donors (Fig. 7C). DEGs of female resilient donors were related to mitochondrial processes, metallothionein and interferon signaling (Fig. 7D), while the DEGs of males did not belong to specific GO or pathways. Furthermore, when using GSEA on the complete dataset including sex chromosomes, the results were similar as when combining both sexes as the majority of processes were either higher expressed in both sexes, such as gene sets related to mitochondria (Fig. 7F; Additional file 5), or more highly expressed in both sexes in AD, like the TYROBP pathway. This was also confirmed with IHC, in which in both sexes mitochondrial MT-CO1 levels pointed into the same direction (Fig. 7G–J). This suggests that on gene-set level mitochondrial genes were more highly expressed in both sexes, although these processes might be more active in female resilient donors, as individual genes such as *MT-CO1*, *MT-CO2*, *MT-CO3* or *MT-CYB* are DEGs in females. Likewise, MT-I/II protein levels and expression of gene sets related to MT were only significantly higher in females. Interestingly, there were some gene sets that were more highly expressed in either male or female resilient donors (Fig. 7F). These included gene sets related to translation, autophagy, and heats shock proteins (heat shock factor 1 activation and cellular response to heat stress) which were more highly expressed in male resilient compared to male AD donors and in female AD compared to male resilient donors. Conversely, processes related to interferon signaling were more highly expressed in the female resilient donors but not in the male donors. Removing the sex chromosomes from the analysis made it possible to directly compare female and male resilient subgroups, which pointed to gene sets related to translation and autophagy or interferon signaling that were more highly expressed in the male or female resilient donors, respectively (Fig. 7E; Additional file 5).

## Discussion

In the present study, we have investigated changes in gene expression in the SFG to gain molecular insight in how individuals remain cognitively intact despite the presence of AD pathology. When comparing resilient to AD donors by GSEA, an enrichment of groups of genes related to mitochondria or MT in the resilient donors was observed, while groups of genes related to immune responses, including the *TYROBP* pathway, were more highly expressed in the AD donors. WGCNA resulted in the identification of gene co-expression networks associated with the UPR, mitochondrial and ribosomal functioning that were significantly different between AD and control but not with resilience. For selected transcripts related to mitochondria, MT or HSPs and the UPR, increased expression was confirmed at the protein level by IHC. Finally, we demonstrate sex-specific differences in gene expression in resilient donors, indicating sex-specific resilient mechanisms. These findings suggest distinct differences in gene expression in the SFG between resilient individuals and AD-patients.

We demonstrate higher levels of expression of MT-I/II in the resilient compared to the AD and control donors. Increased MT-I/II expression was not due to differences in the amount of astrocytes or in pathological load in the resilient compared to the AD donors. There are multiple transcripts encoding for MT, of which the major isoforms MT-1 and MT-2 and the minor isoform MT-3 are found in the brain. MT plays an essential role in metal cellular homeostasis and reduction of reactive oxygen species (ROS). Previous studies have shown increased neuronal Cu^2+^ and Zn^2+^ levels in AD, which can bind to Aβ peptides resulting in redox reactions generating ROS. Higher levels of Cu^2+^ and Zn^2+^ also result in increased generation of the more neurotoxic Aβ oligomers [44] and tau phosphorylation [45]. Notably, lower levels of Aβ oligomers and tau phosphorylation in resilient compared to AD donors have been shown by others [10, 46]. Furthermore, *MT2A* is able to prevent aggregations of Aβ40 and Aβ42 in vitro [47] and overexpression of *MT1* partly ameliorates the phenotype in Tg2576 mice [48]. In addition, astrocytic expression levels of MT-I/II correlated with oxidative DNA damage in astrocytes but not with AD neuropathology in a cohort of aging and AD individuals [39]. Interestingly, increased expression of two metallothionein genes (*MT2A*, *MT1G*) in astrocytes was found in an individual which was resilient to autosomal dominant AD for up to three decades after the expected onset [49]. In this particular individual, reduced levels of pTau were found in the frontal cortex compared to both the hippocampus and occipital cortex with concurrent increased expression of metallothionein in astrocytes in the frontal cortex compared to astrocytes from these other brain regions, as measured by single-nucleus RNA sequencing. Taken together, we hypothesize that in resilient individuals increased metallothionein expression of MT-I/II contributes to a decrease in the amount of ROS, neurotoxic Aβ oligomers and pTau.

Resilient donors had higher expression of mitochondrial genes in the GSEA, which clustered together in the steelblue and saddlebrown modules in the WGCNA analysis. The observation that there are many mitochondrial-derived RNA’s more highly expressed in the resilient group may indicate that there are more active mitochondria or a higher total number of mitochondria in resilient donors compared to AD donors. However, controlling for the amount of pTau pathology negated significance for the enrichment of genes related to mitochondria. This might suggest that higher expression of these genes help to maintain cognition as pathology progresses, up to a certain tipping point. By using IHC, significantly higher levels of MT-CO1 were found outside the soma versus inside the soma in resilient compared to control donors. This points to either a change in mitochondrial activity or an increase in the number of mitochondria that are transported outside of the soma in resilient individuals. Increased levels of mitochondrial proteins is in line with previous research, in which higher protein levels of mitochondrial complex 1 were associated with resilience, after correcting for cognitive decline and AD pathology in a large community-based cohort [50]. Together, these results point to better maintenance of mitochondrial function in resilient donors compared to AD patients.

In this context it is noteworthy that the gene expression changes observed here provide support for the idea that the general cellular homeostasis might be better maintained in the resilient compared to the AD donors. The saddlebrown module contains genes that are not only related to mitochondria, but also to ribosomal function. In AD, mitochondrial dysfunction and impairments in ribosomal function are well documented [51, 52] and have been linked to for example increased levels of ROS. Moreover, the black module containing genes related to HSPs and the UPR was significantly different between the AD and control group. Likewise, using IHC, HSP70 and XBP1s levels were also higher in the AD group compared to the control and resilient groups or only the resilient group, respectively. Activation of the UPR in AD is in line with previous research, as markers for UPR activation related to the PERK-eIF2α and IRE1-XBP1s cascades were higher in post-mortem human brain tissue of AD patients [53–56]. Some HSP70 chaperones have also been linked to the UPR [57]. Furthermore, lower levels of XBP1s by IRE1 deletion has restored learning deficits in AD animals [58], while overexpression of XBP1s stabilizes amyloid precursor protein (APP) expression and binds promotors of γ-secretase complex and genes related to APP metabolism, trafficking and processing [59]. In addition, a polymorphism in the XBP1 promoter is a risk factor for AD [60]. Thus, the lower levels of XBP1s in the resilient donors likely helps to reduce pathology. The initial UPR is activated as a result of endoplasmic reticulum (ER) stress induced by Aβ and tau depositions and is thought to be beneficial, while in advanced stages of AD pathology this response becomes maladaptive under chronic ER stress, increasing neuroinflammation and neurodegeneration. It is conceivable that in resilient individuals this response does not become maladaptive. Collectively, these processes may help to maintain general cellular homeostasis, supporting the hypothesis that cellular health is maintained better in resilient donors despite the presence of AD pathology.

In line with previous research, alterations in gene expression related to microglia were different in resilient compared to AD donors. In the AD donors, there was an enrichment of gene sets related to the *TYROBP* signaling pathway and the innate and adaptive immune system. Furthermore, genes belonging to the pink module, identified in the WGCNA, were related to microglia and neuroinflammation and were more highly expressed in the AD compared to the resilient donors. Others have shown altered microglial states based on markers such as CD68 or TYROBP, which were either increased near plaques [8], decreased near tangles [38] or overall decreased in post-mortem tissue of resilient donors compared to AD patients [5, 7, 61]. In AD animal models, a reduction of TYROBP rescued cognitive deficits and was linked to reduced microglia recruitment and reduced expression of genes associated with a DAM-like phenotype [62]. Taken together, these results suggest that in resilience, microglia are able to more effectively phagocytose Aβ without shifting to a more pathological state in which they increase the release of pro-inflammatory cytokines. Our results substantiate the overall lower levels of immune activation in resilient donors compared to AD. The enrichment of these immune processes in AD compared to resilience are in part negated by controlling for the amount of pTau pathology, which may suggest that with increased pTau levels neuroinflammation is increasing as well.

We have identified a module of genes, the cyan module, related to synaptic signaling, of which the eigengenes showed a trend to be lower in the AD group compared to the control group, while this was not the case for the resilient group. Interestingly, one of the hub genes of this module was synaptophysin, a membrane protein specifically associated with presynaptic vesicles. Others have shown increased levels of synaptophysin in post-mortem tissue of resilient donors [5, 6], further substantiating that synaptophysin and the genes belonging to the cyan module are associated with resilience. Furthermore, we found that the genes belonging to the module midnightblue were more highly expressed in the resilient group compared to the AD group. The genes in this cluster were related to dendritic spines, which have previously been shown to have a different morphology in resilient donors compared to control and AD cases [4]. Contradictory to other studies [28, 63], we found that specific growth factors, such as NRN1, and the abundance of inhibitory cells, were lower in the resilient donors. Mathys et al. [28] showed that inhibitory cells, and the LAMP5-RELN inhibitory subtype, were associated with resilience. While we cannot accurately deconvolute our data to the level of these subtypes [26], we did find a lower proportion of inhibitory cell types. On the other hand, we did find overlap between the 5 DEGs that were found between resilient and AD excitatory neurons in the same dataset, which pointed in the same direction in our dataset. The contradictory results of the differences in proportion of inhibitory neurons could be attributed to the fact that there were differences in the amount of donors used, as Mathys et al. [28] used 427 individuals. Besides, cell-type proportions should ideally be confirmed with a benchmark, such as IHC [64]. Furthermore, the concept of resilience can be seen as a continuum, in which one is able to maintain cognition while pathology progresses up to a certain tipping point. The resilient donors in the current study could be more advanced in the disease process as some interneurons are possibly lost, which occurs in AD patients [65]. This could also explain that the results of the excitatory neurons point in the same direction. Thus, different resilient mechanism might become active depended on the pathological load or disease progression. Finally, differences in brain region or techniques could play a role, as Hurst and colleagues [63] used proteomics and different cortical areas. Increased expression of genes related to synaptic transmission and cellular energy metabolism in resilient donors suggest a compensatory mechanism to preserve cognition despite the presence of AD neuropathology. The increased cellular energy metabolism observed in the current study and by others may be related to the observed maintenance of glucose metabolism in resilient individuals compared to demented individuals using FDG-PET [66]. Taken together, these results suggest that maintenance of synaptic signaling or integrity might be a response in resilient donors to maintain cognition when facing AD neuropathology.

Our data indicate possible sex-dependent resilient mechanisms. While our main findings are upregulated in both sexes, including changes in MT and mitochondrial genes, expression levels are higher in the female donors. Downregulated genes, related to glial cells like the TYROBP pathway, were similar between males and females. Furthermore, we identified sex-specific changes in males related to autophagy and in females related to interferon signaling. Recently, it was suggested that there may be a sex-dependent genetic background in resilience [43] and that markers related to estrogenic, androgenic and neuronal activity differed between sexes in cognitively intact elderly [67]. From this we conclude that there could be sex-dependent mechanisms to maintain cognition.

Interestingly, we have identified a SNORD114-6 in our bulk RNA-seq. data, which was significantly upregulated in the resilient group. While this ncRNA could potentially be upstream of molecular changes related to resilience, we were not able to validate this finding in a subset of our donors with qPCR nor in situ hybridisation. Nevertheless, SNORD114-6 remains an interesting target as it is able to guide 2′-O-methylation on mRNAs and thereby influence alternative splicing and regulate gene expression. It belongs to a major cluster located on the 14q32.2 locus and is maternally expressed, together with the maternally expressed genes (MEGs) MEG3, MEG8, MEG9 and SNORD112 [68]. The best known snoRNAs are SNORD115-SNORD116, which are involved in neurodevelopment disorders such as Prader-Willy syndrome or Angelman syndromes. While similar functions for SNORD113-SNORD114 are less well studied, they have been linked to depression [69], autism [70] and were recently shown to be increased in extracellular vesicles in AD [71]. Sequencing and annotating human snoRNA’s is challenging due to their complex structure [72] and due to quantification errors as the majority of snoRNAs are embedded in introns, causing their reads to be discarded or assigned to the host gene [73]. Current datasets investigating ncRNAs related to resilience did not pick up snoRNAs [74, 75] and specifically designed platforms for detecting snoRNAs did not pick up SNORD114-6 [76, 77]. This hampers the ability to replicate our findings in other datasets. Whether SNORD114-6 is specifically induced in resilience to AD should be investigated with platforms specifically designed to pick up snoRNAs.

Notably, we found that all our potential resilient donors had fewer pathological comorbidities than expected, based on the amount of comorbid pathology often found in AD or aged individuals. Reduced amounts of TDP-43, hippocampal sclerosis and Lewy bodies were found in post-mortem tissue of resilient compared to AD donors [11, 12, 78], while generally in more than half of AD cases TDP-43 was present [79]. Furthermore, in the present study there was a trend towards more AD pathology in the SFG in AD donors compared to the resilient cases. This suggests that the spread of AD pathology throughout the brain, based on Braak and Thal, might be similar between AD and resilient cases while the local neuropathological load could be different, as was recently suggested to be the case in centenarians [80]. Importantly, CERAD scores, albeit not significant, were higher in the AD groups, indicating that there could be more neuritic plaques in the AD donors compared to the resilient donors, which has previously been demonstrated by others [5]. Yet, when controlling for the amount of pathology by using the quantified amounts of pathology as a covariate in our analysis, we were still able to confirm that genes related to MT and the *TYROBP* pathway were more highly expressed in the resilient or AD group, respectively. Likewise, expression of genes encoding for MT-I/II did not correlate with AD pathology. Remarkably, after a careful donor selection there were only 6 DEGs between the AD and resilient group. This observation indicates that in our data, cognitive differences related to resilience have a much smaller effect on gene expression in the SFG than differences in pathology, as there were large differences in both the AD and resilient group compared to control. However, others have found larger differences in gene expression relate to cognition [81] or to resilience [15], using larger datasets. Interestingly, some of the genes identified by Mostafavi et al.[81], which were positively correlated with cognition and reduced Aβ42 in vitro, such as PLXNB1, are downregulated in both AD and resilient donors. Thus, it might be that in resilient donors different mechanisms are activated to maintain cognition as other genes related to cognition are downregulated as part of the disease process. Whether the changes that we have found here are compensatory or could also reduce the amount of AD pathology remains to be further elucidated.

There are some limitations to the current study. After carefully selecting resilient donors using the brain collection of the NBB (n = 2242), 12 individuals fitted our stringent inclusion criteria. The relatively small sample size might be an explanation for the few DEGs we found between the resilient and AD groups. Similar studies investigating resilience to AD with a similar number of resilient donors using proteomics [82] or epigenetics [83] have reported changes in cellular detoxification and repair mechanisms linked to, amongst others, HSPB1 and found that the largest epigenetic changes in resilience are in excitatory neurons and microglia. Others have shown larger differences related to cognition or resilience to AD at the transcriptomic [15, 81] or proteomic level [50, 84, 85] using larger datasets. Nevertheless, our data further corroborates the changes that have previously been found, such as changes in mitochondria, maintenance of cellular health and possible synaptic changes and provides new data on expression of MTs and the UPR. It may be possible that larger differences between resilient individuals and AD patients are found at the proteomic than at the RNA level, which has been shown by comparing co-expression modules from RNA-seq. and proteomics [86]. Finally, to determine whether a donor is cognitively intact, the CDR or GDS was used to determine cognition in the final stage of life. By not being able to measure cognition longitudinally it might be possible that donors classified here as a resilient were already suffering from cognitive decline, albeit not close to clinically relevant levels.

In summary, we provide evidence for changes in gene expression in the SFG that might be related to resilience. The most profound changes include increased expression of genes related to MT, mitochondria and HSPs and the UPR, which were confirmed at the protein level. We also demonstrate putative sex-specific resilient mechanisms and co-expression networks related to cellular health which might contribute to resilience. Taken together, we hypothesize that in face of AD pathology resilient individuals are able to maintain cellular health and increase MT signaling as a possible neuroprotective mechanism.

## Supplementary Information

**Additional file 1.** DEG and GSEA results.

**Additional file 2.** DEGs of AD excitatory neurons versus resilient excitatory neurons of Mathys et al.

**Additional file 3.** The results of the WGCNA analysis, including module membership, GO and hub genes.

**Additional file 4.** IHC of pPERK.

**Additional file 5.** DEG and GSEA results of the sex-specific analysis.

## Data Availability

The RNA-sequencing data is available through Gene Expression Omnibus with accession number GSE261817.

## Abbreviations

AD: Alzheimer’s disease
Aβ: Amyloid beta
APP: Amyloid precursor protein
ApoE: Apolipoprotein E
BDNF: Brain-derived neurotrophic factor
CDR: Clinical dementia rating
CERAD: Consortium to Establish a Registry for Alzheimer's Disease
cIOD: Corrected integrated optical density
DAGLA: Diacylglycerol Lipase Alpha
DEG: Differential expressed gene
FFPE: Formalin-fixed paraffin-embedded
GO: Gene ontology
GSEA: Gene set enrichment analysis
GFAP: Glial fibrillary acidic protein
GDS: Global Deterioration Scale
GRIN1: Glutamate ionotropic receptor NMDA type subunit 1
HSPSA1A: Heat shock 70 kDa protein 1
HSP70: Heat shock protein 70
HSPs: Heat shock proteins
HS: Hippocampal sclerosis
IHC: Immunohistochemistry
Iba1: Ionized calcium-binding adapter molecule 1
LRRK2: Leucine-rich repeat kinase 2
MEG: Maternally expressed gene
LBs: Lewy bodies
LATE-NC: Limbic-predominant age-related TDP-43 encephalopathy
LAMP5: Lysosomal Associated Membrane Protein Family Member 5
MT1M: Metallothionein 1M
MT: Metallothionein
MT1G: Metallothionein 1G
MT2A: Metallothionein 2A
MT-I/II: Metallothionein I–II
MT-CYB: Mitochondrially Encoded Cytochrome B
MT-CO1: Mitochondrially encoded cytochrome c oxidase I
MT-CO2: Mitochondrially encoded cytochrome c oxidase II
MT-CO3: Mitochondrially encoded cytochrome c oxidase III
MEF2C: Myocyte Enhancer Factor 2C
NBB: Netherlands Brain Bank
NRN1: Neuritin 1
OD: Optical density
pPERK: Phosphorylated protein kinase R (PKR)-like endoplasmic reticulum kinase
pTau: Phosphorylated tau
PLXNB1: Plexin B1
PMI: Post-mortem interval
PCA: Principal component analysis
ROS: Reactive oxygen species
RELN: Reelin
RIO: Region of interest
RT: Room temperature
SNORD114-6: Small Nucleolar RNA, C/D Box 114-6
SST: Somatostatin
SYK: Spleen tyrosine kinase
SFG: Superior frontal gyrus
STX4: Syntaxin4
TDP-43: TAR DNA-binding protein 43
TREM2: Triggering receptor expressed on myeloid cells 2
TYROBP: TYRO protein tyrosine kinase-binding protein
UPR: Unfolded protein response
vst: Variance stabilizing transformation
VIP: Vasoactive Intestinal Peptide
WGCNA: Weighted gene co-expression network analysis
XBP1s: X-box binding protein 1, spliced
