# Correction: Gene-expression profiling of individuals resilient to Alzheimer’s disease reveals higher expression of genes related to metallothionein and mitochondrial processes and no changes in the unfolded protein response

**DOI:** 10.1186/s40478-025-01931-2

**Published:** 2025-02-20

**Authors:** Luuk E. de Vries, Aldo Jongejan, Jennifer Monteiro Fortes, Rawien Balesar, Annemieke J. M. Rozemuller, Perry D. Moerland, Inge Huitinga, Dick F. Swaab, Joost Verhaagen

**Affiliations:** 1 Department of Neuroregeneration, Netherlands Institute for Neuroscience, Royal Netherlands Academy of Arts and Sciences, Meibergdreef 47, Amsterdam, 1105 BA The Netherlands; 2 Epidemiology and Data Science, Amsterdam UMC Location University of Amsterdam, Meibergdreef 9, Amsterdam, 1105 AZ The Netherlands; 3 Amsterdam Public Health Methodology, Amsterdam, The Netherlands; 4 Amsterdam Infection and Immunity, Inflammatory Diseases, Amsterdam, The Netherlands; 5 Department of Neuropsychiatric Disorders, Netherlands Institute for Neuroscience, Institute of the Royal Netherlands Academy of Arts and Sciences, Meibergdreef 47, Amsterdam, 1105 BA The Netherlands; 6 Department of Pathology, Amsterdam Neuroscience, Amsterdam UMC - Location VUmc, Amsterdam, The Netherlands; 7 Department of Neuroimmunology, Netherlands Institute for Neuroscience, Institute of the Royal Netherlands Academy of Arts and Sciences, Meibergdreef 47, Amsterdam, 1105 BA The Netherlands; 8 Center for Neuroscience, Swammerdam Institute for Life Sciences, University of Amsterdam, Amsterdam, The Netherlands; 9 Center for Neurogenomics and Cognitive Research, Neuroscience Campus Amsterdam, VU University, Boelelaan 1085, Amsterdam, 1081 HV The Netherlands

**Acta Neuropathologica Communications (2024) 12:68**

10.1186/s40478-024-01760-9

The original publication of this article [1] contained several incorrect values in Table 1. These errors have been amended, the incorrect and correct version of Table 1 are shown in this correction article. The original article has been updated.

Incorrect Table 1.

**Table 1:** Summary of key demographics of matched groups

	Control	AD	Resilient	*P*
N	12	12	11	
Sex	6 M/6F	6 M/6F	5 M/7F	0.895
Age	82.2 ± 10.6	82.0 ± 9.6	87.7 ± 8.2	0.280
pH	6.6 ± 0.3	6.5 ± 0.3	6.4 ± 0.2	0.393
PMI	6.0 ± 1.8	5.1 ± 1.0	6.4 ± 1.7	0.141
CDR/GDS	≤ 0.5 / 1–2	= 3 / 7	≤ 0.5 / 1–2	
ApoE – ε4	3+/9-	7+/5-	5+/6-	0.251
RIN	8.0 ± 0.8	8.1 ± 0.8	7.4 ± 0.6	0.066
BW	1162.8 ± 119.9	1088.7 ± 135.0	1196.4 ± 102.5	0.103
CERAD	0.1 ± 0.3	2.6 ± 0.7	1.7 ± 0.8	< 0.0001
Braak	1.3 ± 0.7	5.5 ± 0.8	4.3 ± 0.9	< 0.0001
Thal	0.7 ± 0.7	4.7 ± 0.8	3.5 ± 0.5	< 0.0001
TDP-43 (NC-LATE)	0	0	0.1	0.336
α-syn	0	0	0	
HS	0	1	0	0.358
Vascular pathology	0	2	1	0.326

Correct Table 1.

**Table 1:** Summary of key demographics of matched groups

	Control	AD	Resilient	*P*
N	12	12	11	
Sex	6 M/6F	6 M/6F	5 M/7F	0.895
Age	82.2 ± 10.6	82.2 ± 9.9	87.7 ± 8.2	0.274
pH	6.6 ± 0.3	6.5 ± 0.2	6.4 ± 0.2	0.092
PMI	6.0 ± 1.8	5.2 ± 1.0	6.4 ± 1.7	0.155
CDR/GDS	≤ 0.5 / 1–2	= 3 / 7	≤ 0.5 / 1–2	
ApoE – ε4	3+/9-	7+/5-	5+/6-	0.334
RIN	8.0 ± 0.8	8.1 ± 0.8	7.4 ± 0.6	0.066
BW	1162.8 ± 119.9	1088.7 ± 135.0	1196.4 ± 102.5	0.103
CERAD	0.1 ± 0.3	2.8 ± 0.6	1.5 ± 0.8	< 0.0001
Braak	1.3 ± 0.7	5.5 ± 0.8	4.3 ± 0.9	< 0.0001
Thal	0.7 ± 0.7	4.7 ± 0.8	3.5 ± 0.5	< 0.0001
TDP-43 (NC-LATE)	0	0	0.1	0.336
α-syn	0	0	0	
HS	0	1	0	0.358
Vascular pathology	0	2	1	0.326

