# Supplementary material for: Gene-expression profiling of individuals resilient to Alzheimer's disease reveals higher expression of genes related to metallothionein and mitochondrial processes and no changes in the unfolded protein response
Source: Acta Neuropathol Commun. 2024 Apr 25;12:68. doi: 10.1186/s40478-024-01760-9 (PMC11046840; doi:10.1186/s40478-024-01760-9)
Supplement: Supplementary file 2 — Additional file 2. DEGs of AD excitatory neurons versus resilient excitatory neurons of Mathys et al. [file 40478_2024_1760_MOESM2_ESM.docx]

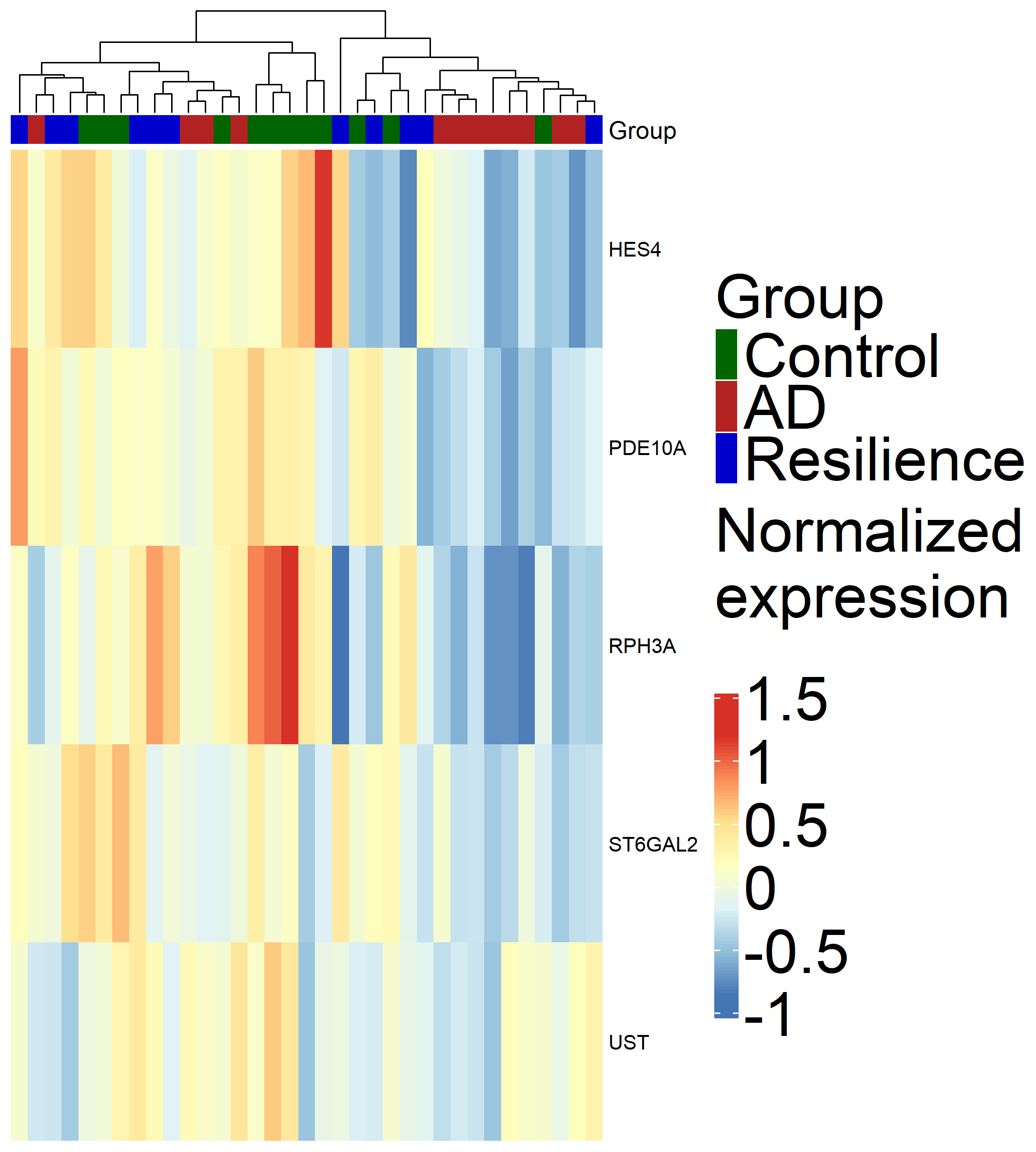


**Additional file 2. Expression levels of DEGs of AD excitatory neurons versus resilient excitatory neurons of Mathys et al.**

A heatmap showing the normalized expression of the 5 DEGs between excitatory neurons of resilient and AD cases, as identified by Mathys et al. The majority of these genes are here downregulated in the AD cases as well, albeit not significant.
