# Supplementary material for: Gene-expression profiling of individuals resilient to Alzheimer's disease reveals higher expression of genes related to metallothionein and mitochondrial processes and no changes in the unfolded protein response
Source: Acta Neuropathol Commun. 2024 Apr 25;12:68. doi: 10.1186/s40478-024-01760-9 (PMC11046840; doi:10.1186/s40478-024-01760-9)
Supplement: Supplementary file 4 — Additional file 4. IHC of pPERK. [file 40478_2024_1760_MOESM4_ESM.docx]

**
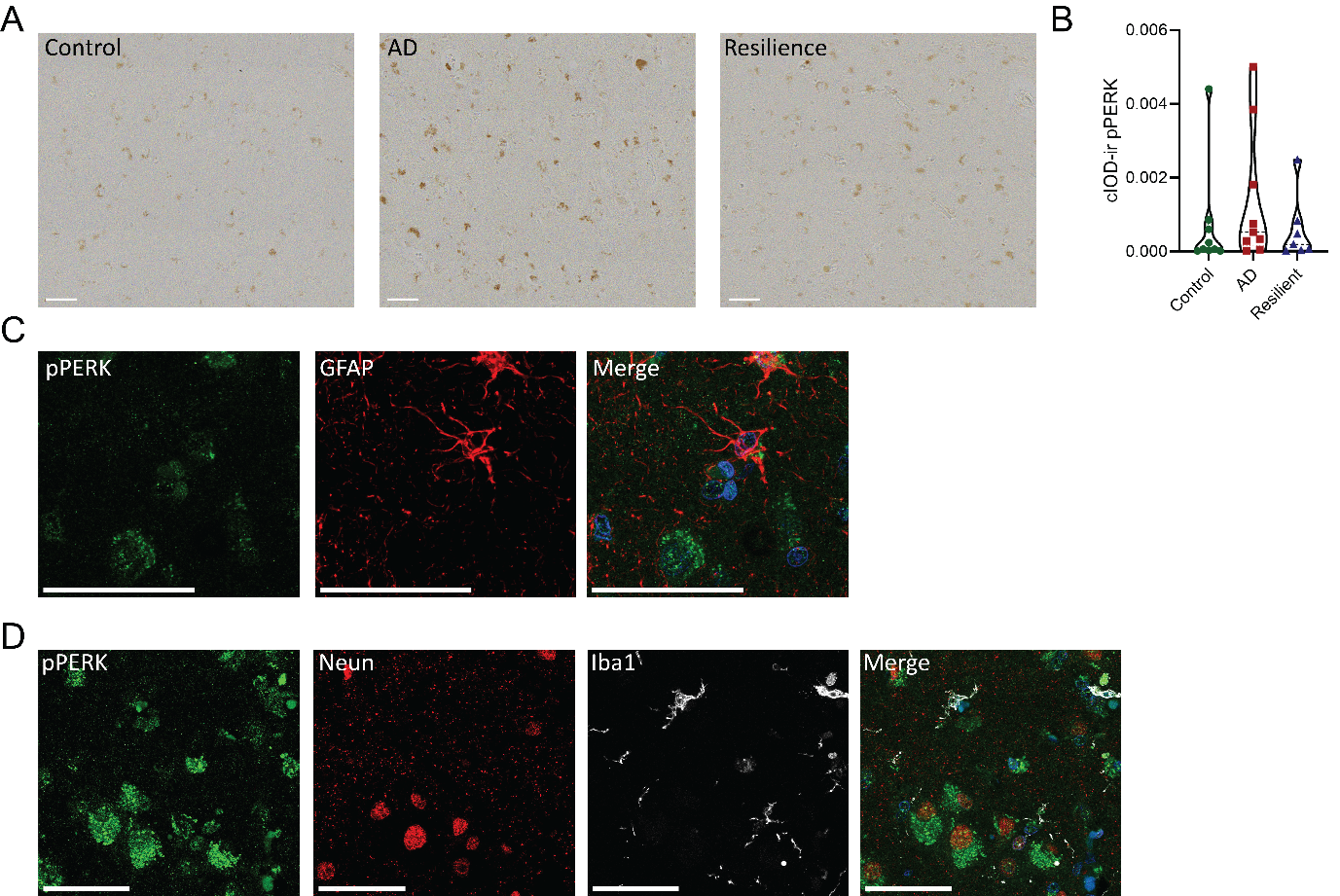
**

**Figure 6. Validation of pPERK**

(A). Representative images of pPERK levels in the different groups. (B) Quantification of pPERK immunoreactivity (pPERK-ir), which was not significantly different between the groups. (C-D) Fluorescent IHC stains show that pPERK (green) is present in GFAP-positive astrocytes (red), NeuN-positive neurons (red) and in Iba1-postive astrocytes (white). All scale bars are 50 µM.
